# Geographical distribution and prevalence of podoconiosis in Rwanda: a cross-sectional country-wide survey

**DOI:** 10.1016/S2214-109X(19)30072-5

**Published:** 2019-03-27

**Authors:** Kebede Deribe, Aimable Mbituyumuremyi, Jorge Cano, Mbonigaba Jean Bosco, Emanuele Giorgi, Eugene Ruberanziza, Ursin Bayisenge, Uwayezu Leonard, Jean Paul Bikorimana, Aniceth Rucogoza, Innocent Turate, Andre Rusanganwa, David M Pigott, Rachel L Pullan, Abdisalan M Noor, Fikre Enquselassie, Jeanine U Condo, Christopher J L Murray, Simon J Brooker, Simon I Hay, Melanie J Newport, Gail Davey

**Affiliations:** a Wellcome Trust Brighton and Sussex Centre for Global Health Research, Brighton and Sussex Medical School, Brighton, UK; b School of Public Health, Addis Ababa University, Addis Ababa, Ethiopia; c Malaria and Other Parasitic Disease Division, Rwanda Biomedical Center–Ministry of Health, Kigali, Rwanda; d Department of Disease Control, London School of Hygiene & Tropical Medicine, London, UK; e Lancaster Medical School, Faculty of Health and Medicine, Lancaster University, Lancaster, UK; f Byumba Hospital, Kibali, Rwanda; g Heart and Sole Africa in Ruhengeri, Musanze, Rwanda; h National Reference Laboratory, Rwanda Biomedical Center, Kigali, Rwanda; i Institute of HIV/AIDS, Disease Control and Prevention Department, Rwanda Biomedical Center, Kigali, Rwanda; j World Health Organization, Rwanda Country Office, Kigali, Rwanda; k Institute for Health Metrics and Evaluation, University of Washington, Seattle, WA, USA; l Kenya Medical Research Institute–Wellcome Trust Collaborative Programme, Nairobi, Kenya; m Centre for Tropical Medicine and Global Health, Nuffield Department of Clinical Medicine, University of Oxford, UK; n Bill & Melinda Gates Foundation, Seattle, WA, USA

## Abstract

**Background:**

Podoconiosis is a type of tropical lymphoedema that causes massive swelling of the lower limbs. The disease is associated with both economic insecurity, due to long-term morbidity-related loss of productivity, and intense social stigma. Reliable and detailed data on the prevalence and distribution of podoconiosis are scarce. We aimed to fill this data gap by doing a nationwide community-based study to estimate the number of cases throughout Rwanda.

**Methods:**

We did a population-based cross-sectional survey to determine the national prevalence of podoconiosis. A podoconiosis case was defined as a person with bilateral, asymmetrical lymphoedema of the lower limb present for more than 1 year, who tested negative for *Wuchereria bancrofti* antigen (determined by Filariasis Test Strip) and specific IgG4 (determined by Wb123 test), and had a history of any of the associated clinical signs and symptoms. All adults (aged ≥15 years) who resided in any of the 30 districts of Rwanda for 10 or more years were invited at the household level to participate. Participants were interviewed and given a physical examination before Filariasis Test Strip and Wb123 testing. We fitted a binomial mixed model combining the site-level podoconiosis prevalence with continuous environmental covariates to estimate prevalence at unsampled locations. We report estimates of cases by district combining our mean predicted prevalence and a contemporary gridded map of estimated population density.

**Findings:**

Between June 12, and July 28, 2017, 1 360 612 individuals—719 730 (53%) women and 640 882 (47%) men—were screened from 80 clusters in 30 districts across Rwanda. 1143 individuals with lymphoedema were identified, of whom 914 (80%) had confirmed podoconiosis, based on the standardised diagnostic algorithm. The overall prevalence of podoconiosis was 68·5 per 100 000 people (95% CI 41·0–109·7). Podoconiosis was found to be widespread in Rwanda. District-level prevalence ranged from 28·3 per 100 000 people (16·8–45·5, Nyarugenge, Kigali province) to 119·2 per 100 000 people (59·9–216·2, Nyamasheke, West province). Prevalence was highest in districts in the North and West provinces: Nyamasheke, Rusizi, Musanze, Nyabihu, Nyaruguru, Burera, and Rubavu. We estimate that 6429 (95% CI 3938–10 088) people live with podoconiosis across Rwanda.

**Interpretation:**

Despite relatively low prevalence, podoconiosis is widely distributed geographically throughout Rwanda. Many patients are likely to be undiagnosed and morbidity management is scarce. Targeted interventions through a well coordinated health system response are needed to manage those affected. Our findings should inform national level planning, monitoring, and implementation of interventions.

**Funding:**

Wellcome Trust.

## Introduction

Podoconiosis is a type of tropical lymphoedema that is caused by long-term exposure to volcanic soils.^1^ The disease occurs among genetically susceptible individuals who do not use proper footwear.^2^, ^3^ The disease is thought to be endemic in 32, mostly tropical, countries^4^ and it is estimated that 4 million people globally are currently living with podoconiosis.^3^ Owing to its high burden of morbidity, those with podoconiosis suffer severe stigmatisation,^5^, ^6^ reduced quality of life^7^ and productivity,^8^ and increased rates of depression.^9^

Limited and inequitable access to morbidity management services for neglected tropical diseases is a cause of growing concern globally.^10^ Podoconiosis is a neglected tropical disease and is common in low-income and middle-income countries.^4^ In these countries, inadequate surveillance systems, dysfunctional community referral systems, and poor awareness are the most common barriers to the provision of podoconiosis care. The overall burden of podoconiosis is largely unknown, even within endemic countries, severely limiting advocacy for intervention and evaluation at the national level.^11^

Reliable and detailed data on the prevalence and distribution of podoconiosis globally are scarce; those available are restricted to scattered endemic communities and case reports,^4^ leaving the actual prevalence within confirmed and suspected endemic areas unknown.^11^ To date, available estimates of podoconiosis largely depend on expert opinion. These estimates have been useful in raising necessary awareness, yet are clearly insufficient for planning national control programmes. A 2018 systematic review^4^ found an absence of prevalence data on podoconiosis in many of the suspected endemic countries.^4^

**Research in context**

**Evidence before this study**

We searched PubMed, Embase, and Popline databases for original studies of prevalence of podoconiosis in Rwanda published between Jan 1, 1970, and Aug 9, 2018, with no language restrictions. We used the search terms “podoconiosis” AND “non-filarial elephantiasis” AND “prevalence”, AND “Rwanda”, and manually searched the reference lists of articles identified. One study on the prevalence of elephantiasis in Rwanda was published in 1976. The study observed cases in special clinics and market-based surveys, and identified 133 elephantiasis cases in special clinics. 20 446 individuals were screened in 14 markets, giving a prevalence of 6·26 per 1000 (range 1·2–16·7 per 1000 per market). To our knowledge, no community-based study has been done to estimate the prevalence of podoconiosis in Rwanda.

**Added value of this study**

This study is the first community-based study to estimate the prevalence of podoconiosis in Rwanda, and is also the first study in Rwanda to use a clinical algorithm to diagnose podoconiosis by excluding other potential causes of lymphoedema. We excluded *Wuchereria bancrofti* antigen and specific IgG4 using the Filariasis Test Strip and Wb123 tests, respectively. We fitted a binomial mixed model combining the site-level podoconiosis prevalence with continuous environmental covariates to estimate prevalence at unsampled locations. Finally, we produced estimates of cases by district, combining our mean predicted prevalence and a contemporary gridded map of estimated population density.

**Implications of all the available evidence**

Despite the relatively low prevalence, our findings show that podoconiosis is widely distributed throughout Rwanda. Estimating the number of cases and the geographic distribution of podoconiosis is important for local advocacy, policy, and programming of podoconiosis interventions in Rwanda. The methods we used are likely to inform mapping of the global distribution of podoconiosis.

Over the past decade, the global health community has recognised the need to accurately estimate podoconiosis burden within endemic countries.^11^ In Rwanda, only one study,^1^ done in 1976, has documented the prevalence of visible lymphoedema in a market-based analysis. This work estimated a prevalence of 0·63% of visible lymphoedema across Rwanda. Although this study is a landmark paper in podoconiosis research, it has two notable limitations. First, it was based on the observation of visible lymphoedema and did not exclude other potential causes of lymphoedema.^1^ Second, the study's target population included only those who attended markets, leaving out the possibility of additional cases. No additional research has been done to estimate the prevalence of podoconiosis in Rwanda. Updated and robust estimates of the distribution and number of cases of podoconiosis in Rwanda will help to identify endemic areas that require intervention and exclude areas without risk of the disease. It is also important to define the number of people with podoconiosis in the country, to enable effective planning and careful use of resources.

This study is part of a larger initiative to develop the global atlas of podoconiosis.^11^ We aimed to determine the prevalence and geographical distribution of podoconiosis within all districts of Rwanda among the adult population (aged ≥15 years) and to estimate the number of podoconiosis cases by district across the country. We did a nationwide survey and used the observed prevalence at selected sites to construct a predictive prevalence model for the entire country. In presenting the results, we address the essential first step of the WHO action plan of elimination of neglected tropical diseases: mapping the geographical distribution of such diseases.^12^

## Methods

### Study design and participants

We did a population-based cross-sectional survey on podoconiosis targeting all 30 districts in Rwanda. In July, 2017, trained community health workers did a census of the communities within the selected sectors (a sector being the smallest administrative unit in Rwanda). In November, 2017, expert clinical diagnostic teams verified all suspected cases of lymphoedema (listed by community health workers) within these sectors. Rwanda is a densely populated, landlocked country of about 26 000 km^2^ in central eastern Africa. The country has a population of 12·1 million people (as of 2018)^13^ and is administratively divided into five provinces, 30 districts, and 416 sectors.^13^ Important country indicators include maternal mortality of 210 per 100 000 livebirths and child mortality of 50 per 1000 livebirths.^14^ The average life expectancy is 66·6 years.^13^

All adults (aged ≥15 years) who lived in any of the 30 districts were included in the study. Exclusion criteria were terminally ill patients who could not respond to the interview and patients with a mental health condition that would make interviewing difficult and results unreliable.

Ethical approval was obtained from the Rwanda National Ethics Committee and the Brighton and Sussex Medical School Research Governance and Ethics Committee, Brighton, UK. Written informed consent was obtained from all respondents, except for illiterate respondents who provided their thumbprint and a signature from a literate witness. Individuals younger than 18 years provided assent and a parent or guardian provided written consent.

A summary of the protocol is available in the appendix.

### Procedures

Between June 12, and July 28, 2017, 282 trained community health workers did a census of the communities within the selected sectors. They registered residence, sex, and age, and recorded the presence of leg swelling of any type. All individuals with swelling of one or both lower limbs were documented as suspected cases during an exhaustive house-to-house census and case listing. Community volunteers were provided with case definitions and pictures of podoconiosis-related morbidity to identify the suspected cases.

Between Nov 19, and Dec 6, 2017, expert clinical diagnostic teams verified all suspected cases of lymphoedema (listed by community health workers) within the randomly selected sectors, on the basis of procedures previously used in Cameroon.^15^ Each team included four health workers, a medical doctor, a nurse, a laboratory technician, and a team leader. To ensure effective community engagement, members of the team were recruited from the targeted sectors. Questionnaires were translated into Kinyarwanda language and data were collected using the LINKS software package (version 1.4.2; Secure Data Kit, Atlanta, GA, USA) installed onto Android smartphones.^16^ Individual data on age, sex, education, occupation, place of residence, shoe wearing, and foot hygiene practices were recorded, as were household data on water, sanitation, and hygiene. Geographic coordinates from surveyed communities were taken using smartphones. Data collectors could not proceed to the next question without completing all required fields. We used automated skip patterns to maintain the quality of the data.

Each patient with suspected podoconiosis underwent a complete physical examination in a private room at the nearest public health centre. We used data collection methods used in previous similar surveys done in other countries.^15^, ^17^ Key questions were age at onset of swelling, family member (living or dead) with history of leg swelling, type of swelling (ascending or descending), and self-reported chronic illness, such as heart disease, kidney disease, or diabetes. Ascending swelling refers to swelling that starts from the foot and progresses up the leg; descending swelling is swelling that starts from the upper leg or groin area and progresses down. Suspected patients were also asked about previous clinical diagnoses of known causes of lymphoedema (such as congenital disorders, leprosy, and postoperative lymphoedema) and about the co-occurrence of swelling in other parts of the body, such as the hands, face, and scrotum (hydrocele).

### Differential diagnosis with lymphatic filariasis

All lymphoedema cases were screened for circulating *Wuchereria bancrofti* antigen, using Filariasis Test Strips, and circulating specific IgG4 antibody, using Wb123 tests. Screening was done by trained laboratory technicians according to the manufacturer's instructions.^18^, ^19^ Test results with the individual's unique ID number were recorded both on the card, and on each individual's data questionnaire.

Briefly, for the rapid test, we used positive and negative controls for quality control of test batches before starting the daily activity. The patient's third or fourth finger was cleaned with 70% alcohol and punctured using a sterile lancet. The initial drop of blood was removed using a cotton swab, and sufficient fresh blood was obtained to fill a 75-μl capillary tube. The blood sampled was transferred from the capillary tube to the pad on a Filariasis Test Strip card. The result of each card was read at 10 min exactly. A positive result was two lines, and a negative result was a single line. The tests were repeated if the control line was not shown.

For the detection of IgG4 antibodies against *W bancrofti*, 10-μl capillary blood was transferred from the capillary tube to the pad of a Wb123 card and four drops of assay diluent were dispensed vertically into the square assay diluent well. The result of each Wb123 card was read at 30 min. A positive result was two lines, and a negative result was a single line.

### Modelling of podoconiosis prevalence

The following covariates were used in our analysis in a gridded format (raster datasets): precipitation, day land surface temperature, elevation, enhanced vegetation index, distance from closest waterway, clay content, silt content, night light emissivity, and distance to stable night light (appendix). These factors are associated with podoconiosis and have been included in previous modelling studies.^20^ Details on the source and processing are available in the appendix.

Input grids were resampled to a common spatial resolution of 1 km^2^ using the nearest neighbour approach and clipped to match the geographic extent of a map of Rwanda, and eventually aligned to the map. Raster manipulation and processing was done using the raster package in R v3.3.2 and final map layouts created with ArcGIS software (version 10.5; Esri, Redlands, CA, USA). Geographic coordinates of each community were used to extract estimates from the aforementioned covariates.

### Outcomes

We defined a podoconiosis case as a person residing in the surveyed district for at least 10 years who had bilateral, asymmetrical lymphoedema of the lower limb lasting for more than 1 year, negative Filariasis Test Strip (Alere; Scarborough, ME, USA) and Wb123 tests, and a history of any of the signs and symptoms associated with podoconiosis.^17^ We collected occupational data in precoded categories: government employee, non-government employee, subsistence farmer or fishing, self-employed, full-time student, at home doing housework, unemployed (but able to work), unemployed (unable to work), and retired. Collected data were screened every day by the field supervisors and immediate feedback was given to the enumerators; field team leaders gave overall feedback and supervision. Final assessment of the full database was done after data collection every day to identify inconsistencies and missing items.

We used a system for grading the clinical stages of podoconiosis that had been developed and validated in Ethiopia previously.^21^ The system has five stages: stage 1, swelling reversible overnight (ie, the swelling is not present when the patient first gets up in the morning); stage 2, below-knee swelling that is not completely reversible overnight, with knobs or bumps below the ankle only (if present); stage 3, below-knee swelling that is not completely reversible overnight, with knobs or bumps above the ankle; stage 4, above-knee swelling that is not completely reversible overnight, with knobs or bumps at any location; and stage 5, swelling at any place in the foot or leg, and the ankle or toe joints become fixed and difficult to flex or dorsiflex—these symptoms can be accompanied by apparent shortening of the toes.

### Statistical analysis

The survey used a cluster sampling design. We included all 30 districts of Rwanda. In each district, at least two sectors were randomly selected. The survey was powered to generate prevalence estimates for podoconiosis with a precision of 0·003% at the national level, assuming a conservative prevalence estimate of 42 cases per 100 000 from a surveillance report (M Jean Bosco, Rwanda Biomedical Center–Ministry of Health, personal communication). Under a design effect of 1·6 (calculated from a mapping survey in Cameroon)^15^ and a community participation rate of 80%, we estimated a sample size of 986 376 individuals to be screened from 60 sectors (two sectors per district). To adjust for sector size, sectors were assigned to districts proportional to the number of sectors per district. The adjustment resulted in 80 of 416 sectors being selected, with independent selection in each district. In the selected sectors, a census of all eligible individuals was done. All individuals aged 15 years and older, who had lived in the area for at least 10 years before the survey (to exclude individuals who might have acquired lymphoedema elsewhere) were included.

We described the individual characteristics and prevalence of lymphoedema and podoconiosis with 95% CIs. We estimated district-level prevalence and number of cases across Rwanda using a binomial mixed model, accounting for fixed effects (covariates) and random effects. We chose this method over geostatistical and Bayesian frameworks on the basis of the absence of spatial structure on podoconiosis prevalence, explored by fitting an empirical variogram (appendix). Spatial dependence is a prerequisite for such methods. Our model used the prevalence estimates and covariates for smoothing the prediction. Briefly, let *Y*_i_ denote the random variable associated with the number of positively detected cases of podoconiosis at a community location *x*_i_. We then modelled *Y*_i_ using a binomial mixed model with probability of having podoconiosis *p(x*_i_*)* such that:

(1) $$\log\left\{\frac{p(x_{i})}{1-p(x_{i})}\right\}=\beta_{0}+\sum_{j=1}^{9}j\beta_{j}d_{j}(x_{i})+Z_{i}$$

where the *d*_j_*(x*_i_*)* are georeferenced covariates and *Z*_i_ are independent and identically distributed zero-mean Gaussian variables with variance σ^2^. We fitted the model using the lme4 package (version 3.4) in R software.^22^ Using the approach described by Bates and colleagues,^23^ we tested the presence of residual spatial correlation by generating the 95% CIs for the variogram of the random effects *Z*_i_ under the assumption of spatial independence. As the variogram that was based on the estimated *Z*_i_ using the original data fell within the 95% CIs, we concluded that there was no evidence of residual spatial correlation. Therefore, a binomial non-spatially explicit mixed model was constructed. This model was used to produce continuous predictions of podoconiosis prevalence at 1 km^2^ spatial resolutions. We also developed a probability map of exceeding 0·1% prevalence. For a given location *x*, we obtain

(2) $$\text{Prob}\left\{\hat{\beta_{0}}\sum_{j=1}^{9}\hat{\beta_{j}}d_{j}(x)>\log\left(\frac{0.1\%}{1-0.1\%}\right)\right\}$$

where β_j_ hat is the maximum likelihood estimate of the regression coefficient β_j_ for *j*=0,1,...,9. To compute the probability of exceeding 0·1% prevalence, we used the multivariate Gaussian approximation of the maximum likelihood estimator.^24^

Gridded maps of population density and age structure were obtained from the WorldPop project.^25^, ^26^ We used this gridded population surface to compute the estimates of affected population by pixel, by multiplying prevalence of podoconiosis in 1 km^2^ area with the corresponding population at the same spatial resolution. We used this surface to extract the aggregate number of people with podoconiosis by district.

### Role of the funding source

The funder of the study had no role in study design, data collection, data analysis, data interpretation, or writing of the report. The corresponding author and the last author had full access to all the data and had final responsibility for the decision to submit for publication.

## Results

**Figure 1:**
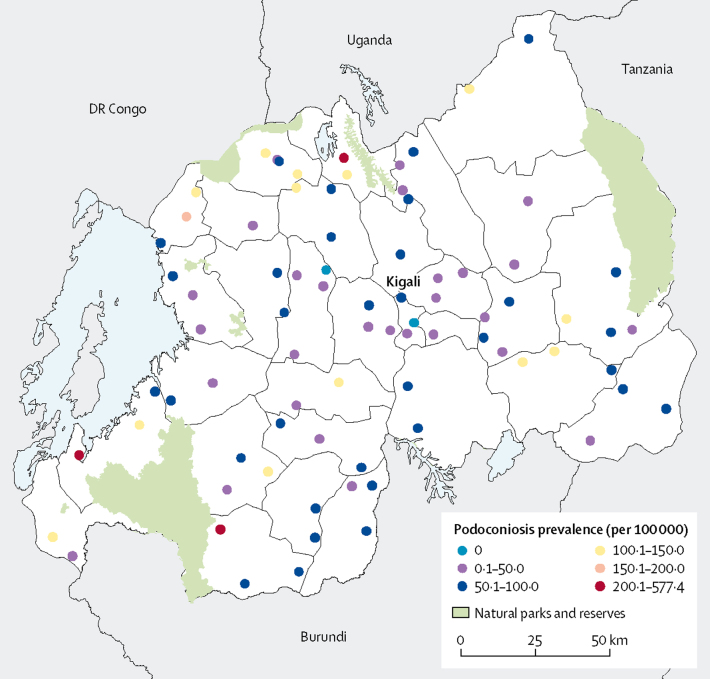
Distribution of surveyed communities and prevalence of podoconiosis across Rwanda

**Figure 2:**
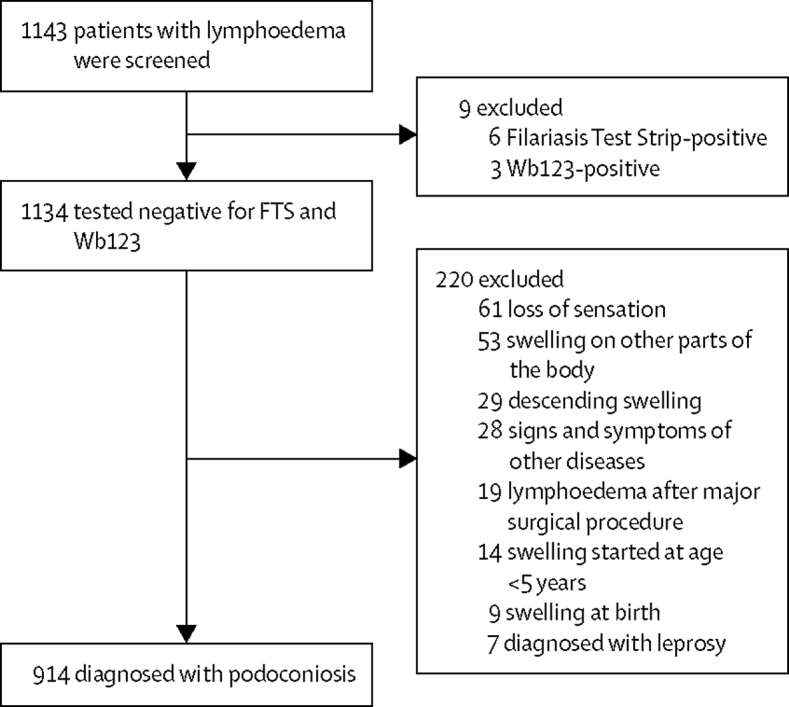
Study profile Podoconiosis was diagnosed via history, physical examination, and disease-specific tests.

Between June 12, and July 28, 2017, 1 360 612 individuals—640 882 (47%) men and 719 730 (53%) women—were screened by community health workers in 80 sectors in 30 districts (figure 1). 1143 individuals with lymphoedema were identified and tested using Filariasis Test Strip and Wb123 tests. 229 (20%) of 1143 were excluded for having non-podoconiosis lymphoedema (figure 2).

914 (80%) individuals with swelling of lower limbs were considered to be podoconiosis cases, yielding a prevalence of 68·5 per 100 000 people (95% CI 41·0–109·7). The median age was 55 years (IQR 43–68) for people with non-podoconiosis lymphoedema and 53 years (40–66) for people with podoconiosis (table 1). 214 (93%) people with non-podoconiosis lymphoedema and 800 (88%) people with podoconiosis reported subsistence farming as their occupation. Most participants reported no formal education and most participants with lymphoedema lived in a household with an earth or sand floor, although the majority were wearing shoes during the interview (table 1).

**Table 1:** Baseline characteristics

		**Non-podoconiosis (n=229)**	**Podoconiosis (n=914)**
Age, years			
	15–24	15 (7%)	56 (6%)
	25–34	17 (7%)	88 (10%)
	35–44	32 (14%)	151 (17%)
	45–54	40 (17%)	184 (20%)
	55–64	54 (24%)	180 (20%)
	65–74	41 (18%)	136 (15%)
	75–84	21 (9%)	95 (10%)
	≥85	9 (4%)	24 (3%)
Median age, years		55 (43–68)	53 (40–66)
Employment			
	Subsistence	214 (93%)	800 (88%)
	Not working	9 (4%)	100 (11%)
	Self-employed	3 (1%)	6 (1%)
	Salaried	3 (1%)	6 (1%)
	Housework	0	2 (<1%)
Sex			
	Male	83 (36%)	276 (30%)
	Female	146 (64%)	638 (70%)
Educational achievements			
	No formal education	155 (68%)	642 (70%)
	Primary	70 (31%)	249 (27%)
	Secondary	1 (<1%)	19 (2%)
	Tertiary	3 (1%)	4 (<1%)
Marital status			
	Single	30 (13%)	155 (17%)
	Married	114 (50%)	432 (47%)
	Divorced	19 (8%)	70 (8%)
	Widowed	66 (29%)	257 (28%)
Type of floor in the household			
	Earth or sand	209 (92%)	837 (92%)
	Ceramic tiles	20 (9%)	64 (7%)
	Dung	0	9 (1%)
	Carpet	0	3 (<1%)
	Vinyl or asphalt strips	0	1 (<1%)
Have you ever worn shoes?			
	Yes	218 (95%)	858 (94%)
	No	11 (5%)	56 (6%)
Median age at wearing shoes, years (IQR)		23 (12–30)	24 (13–30)
Wearing shoes during the interview?			
	Yes	194 (85%)	770 (84%)
	No	35 (15%)	88 (16%)
Type of shoe worn during the interview			
	Hard plastic	99 (43%)	380 (42%)
	Open sandal	69 (30%)	291 (32%)
	Leather	18 (8%)	66 (7%)
	Canvas	6 (3%)	27 (3%)
	Other	2 (1%)	6 (1%)
Family history of leg swelling			
	Yes	91 (40%)	390 (43%)
	No	138 (60%)	524 (57%)
Age at onset of swelling, years		25 (15–39)	21 (15–36)
Source of water			
	Pipe-borne	186 (81%)	633 (70%)
	Borehole or well	14 (6%)	128 (14%)
	River or stream	20 (9%)	120 (13%)
	Pond or stagnant	9 (4%)	33 (4%)
Location of water source			
	Elsewhere	222 (97%)	873 (96%)
	In own yard or plot	0	14 (2%)
	In own dwelling	7 (3%)	27 (3%)
Time taken to go to water source, collect water, and return home, mins		30 (20–60)	30 (20–60)
Podoconiosis disease stage*			
	Stage 1	..	274 (30%)
	Stage 2	..	443 (48%)
	Stage 3	..	169 (18%)
	Stage 4	..	23 (3%)
	Stage 5	..	5 (1%)
Have you experienced an acute attack in the past 6 months?			
	Yes	..	512 (56%)
	No	..	402 (44%)

Data are n (%) or median (IQR).

* Stage 1, swelling reversible overnight. Stage 2, below-knee swelling that is not completely reversible overnight; if present, knobs or bumps are below the ankle only. Stage 3, below-knee swelling that is not completely reversible overnight; knobs or bumps are above the ankle. Stage 4, above-knee swelling that is not completely reversible overnight; knobs or bumps are at any location. Stage 5, swelling at any place in the foot or leg; the ankle or toe joints become fixed and difficult to flex or dorsiflex; these symptoms can be accompanied by apparent shortening of the toes.

**Figure 3:**
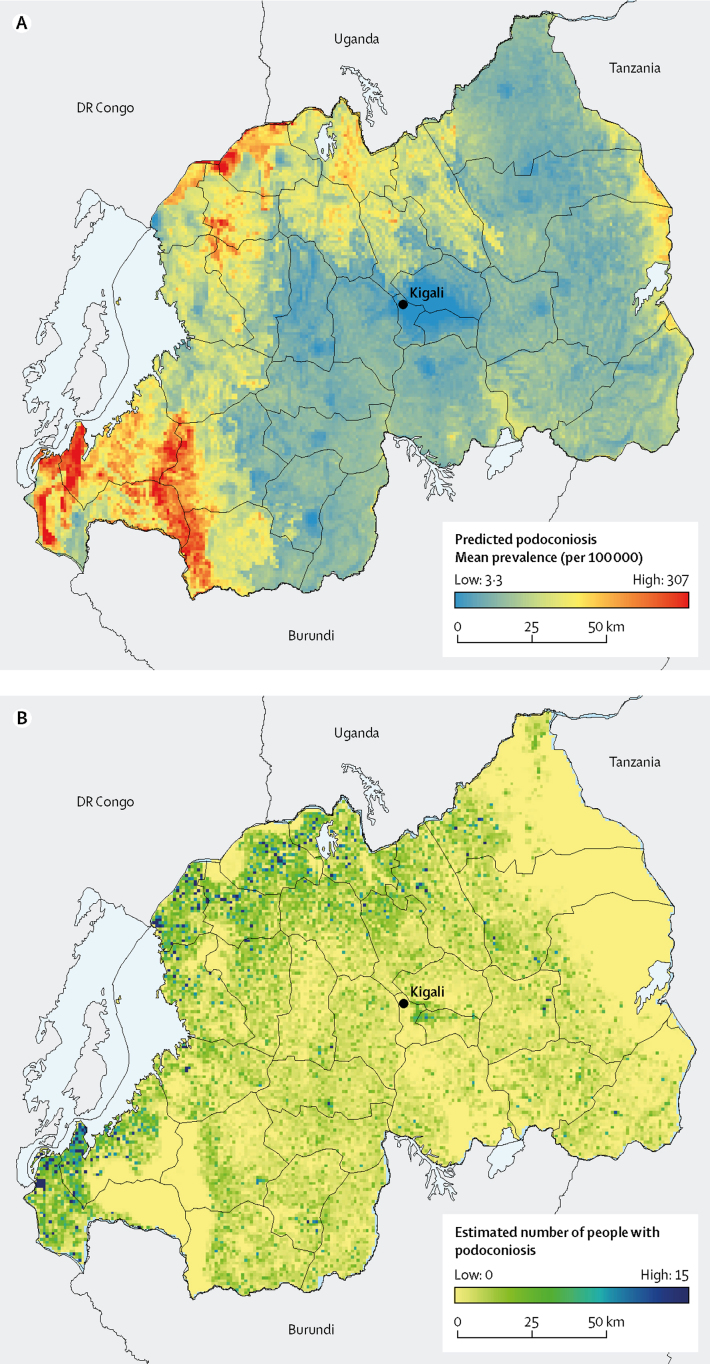
Predicted prevalence of podoconiosis Mean predicted prevalence of podoconiosis (A) and estimated number of people with podoconiosis (B).

The predicted distribution of podoconiosis risk was heterogeneous, varying from 3·3 to 307 per 100 000 (figure 3). The highest prevalence is predicted in restricted areas in the north and southwest, which are also the least populated areas in Rwanda. 17 clusters with prevalence exceeding 1 per 1000 were recorded in 11 districts, mostly in northern and western provinces. The predicted prevalence of podoconiosis was highest in the North and West provinces, where prevalence exceeded more than 70 per 100 000 in most districts. The districts with the highest prevalence were Nyamasheke (119·3 per 100 000) and Rusizi (117·0 per 100 000) in the West province, followed by Musanze Nyabihu, Nyaruguru, Burera, and Rubavu.

**Figure 4:**
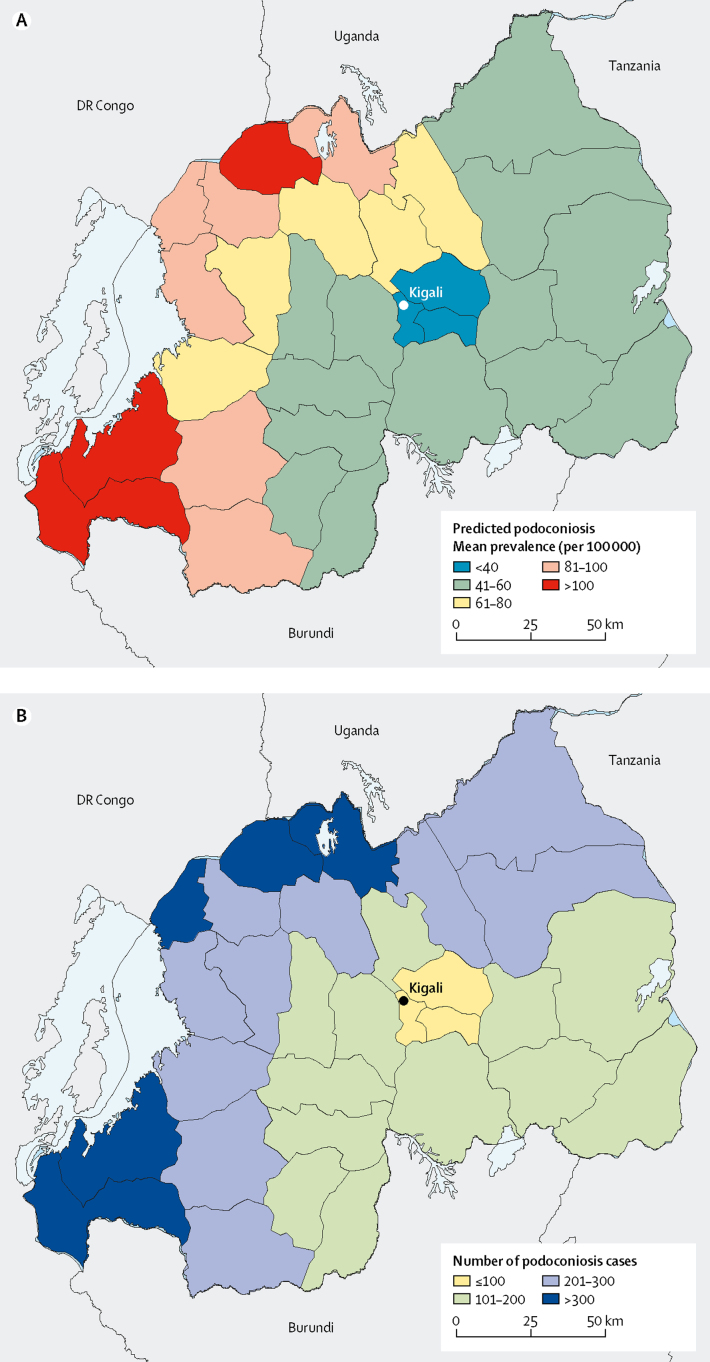
District-level predicted prevalence of podoconiosis Mean predicated prevalence of podoconiosis (A) and estimated number of people with podoconiosis (B).

**Figure 5:**
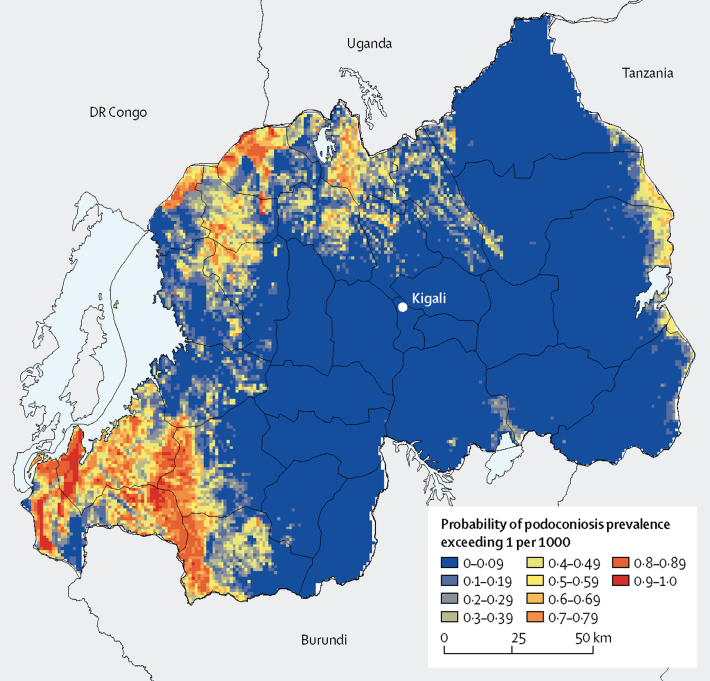
Probability of podoconiosis prevalence exceeding 1 per 1000 people in Rwanda

The highest number of people with podoconiosis was predicted in the West province (2096, 95% CI 1140–3591). In the eastern and northern districts, the prevalence and number of cases is low (figure 4). Nationally, we estimated that 6429 people (95% CI 3938–10 088) were living with podoconiosis (table 2). 27 of 30 districts had at least 100 predicted podoconiosis cases and 15 had more than 200 predicted podoconiosis cases (table 2). Maps displaying the uncertainties around the predicted prevalence and estimates of number of cases are included in the appendix. We also estimated the continuous probability of exceeding 1 case per 1000 across the endemic areas (figure 5). Most of the country had a low probability of exceeding this prevalence, and only a few districts in the north and southwest of the country would potentially exceed that threshold.

**Table 2:** Estimated number of podoconiosis cases by province and district

		**Podoconiosis cases, n**	**95% CI**
East			
	Bugesera	187	129–262
	Gatsibo	237	161–337
	Kayonza	175	120–246
	Kirehe	196	126–291
	Ngoma	194	131–277
	Nyagatare	267	183–380
	Rwamagana	150	105–208
Kigali			
	Gasabo	91	52–164
	Kicukiro	54	29–100
	Nyarugenge	22	10–48
North			
	Burera	313	193–487
	Gakenke	262	161–405
	Gicumbi	290	176–452
	Musanze	317	187–512
	Rulindo	193	127–281
South			
	Gisagara	170	115–242
	Huye	150	100–218
	Kamonyi	160	115–218
	Muhanga	127	77–199
	Nyamagabe	215	127–343
	Nyanza	157	108–221
	Nyaruguru	231	141–362
	Ruhango	175	124–241
West			
	Karongi	241	149–373
	Ngororero	245	148–387
	Nyabihu	246	123–445
	Nyamasheke	449	232–798
	Rubavu	291	165–479
	Rusizi	381	178–725
	Rutsiro	243	146–384
National total		6429	3938–10 088

## Discussion

This study is, to our knowledge, the first nationwide, population-based study of podoconiosis in Rwanda. Our findings suggest that although the prevalence of podoconiosis is low (68·5 estimated cases per 100 000), it is widespread throughout Rwanda, indicating that environmental conditions suitable for the occurrence of podoconiosis are present throughout most of the country. According to our fitted model, 6429 individuals potentially have podoconiosis in Rwanda. We found considerable geographical variability in the prevalence of podoconiosis across the country. The highest prevalence foci for podoconiosis are predicted in the north and southwest districts, although cases are widely distributed across all 30 districts of Rwanda. The number of cases highlights the need to address this issue through a health system response.

The prevalence of podoconiosis in this study is lower than the findings reported in other endemic countries in Africa.^4^ For example, estimated prevalence is 2·73–7·45% in Ethiopia, in Cameroon 0·51–8·08%, and in Uganda 0·10–4·52%.^4^ The lower prevalence of podoconiosis in our study than in other studies done in the aforementioned countries might result from differences in survey design, case definition, or areas targeted for the survey (most studies were done in endemic areas^4^), amongst other things. However, another plausible reason might be a greater use of prevention measures, such as footwear and improved access to water in Rwanda. Over the last two decades, Rwanda has registered impressive economic development.^27^, ^28^ Prevalence of podoconiosis was highest in districts in the north and southwest of the country. Districts in the Kigali province had the lowest prevalence of podoconiosis, despite being highly populated. Kigali is one of the most developed and socioeconomically advanced provinces in the country; thus lower prevalence of podoconiosis is to be expected. This finding suggests that the Ministry of Health in Rwanda should consider district-specific interventions, by prioritising districts with higher numbers of cases.

Previous reports^29^ have highlighted the absence of lymphatic filariasis in Rwanda, but the causes of lymphoedema in the country had not been comprehensively studied until now. Our study indicates that 80% of tropical lymphoedema in Rwanda is due to podoconiosis; this value is high compared with the proportion reported in Cameroon (63%)^15^ and Ethiopia (65%).^30^ This disparity implies that front-line health workers in Rwanda should be given podoconiosis training to increase the index of suspicion for podoconiosis when people present with lymphoedema. Morbidity management services integrated with regular health services should be implemented. Loss of sensation was one of the most frequent clinical features used to exclude podoconiosis. Podoconiosis patients have intact nerves and no loss of sensation in their legs is expected from this condition.^17^ The cause of loss of sensation in our study could be diabetic neuropathy, which is common among patients with diabetes in Rwanda.^31^ However, we cannot exclude undiagnosed leprosy.^32^

Six (0·5%) Filariasis Test Strip tests and three (0·3%) Wb123 tests were positive among the screened lymphoedema cases. None of these individuals was positive for both tests. The six Filariasis Test Strip-positive patients were from five districts: Ngoma in East province, Gasbo in Kigali province, Gisagara and Nyamagabe in South province, and Rusizi in West province. These findings are not the first time that antigen-positive cases have been reported in Rwanda. In a 2008 survey, of 797 individuals surveyed, a single immunochromatographic card test-positive case was found and, through subsequent tests, the person was confirmed to harbour *W bancrofti*. However, subsequent screening of 200 individuals from the same district found no positive cases, confirming the absence of ongoing transmission of lymphatic filariasis in the district.^29^ In another survey done in 13 districts in 2008, two immunochromatographic card test-positive cases were found among 1494 individuals surveyed in two districts.^33^ In our study, we tested individuals with lymphoedema, which increased the odds of identifying lymphatic filariasis-related cases compared with surveying the general population. Given these findings and the specificity^34^ of the tests, our results do not suggest ongoing transmission of lymphatic filariasis in Rwanda. To confirm the results of the Filariasis Test Strip-positive cases, we are working with the national programme to test these individuals using thick blood smear done at night.

Our findings suggest that podoconiosis is a widespread problem in Rwanda and is present in all 30 districts. Prevalence varied from 28·3 per 100 000 (95% CI 16·8–45·5; Nyarugenge, Kigali province) to 119·2 per 100 000 (59·9–216·2; Nyamasheke, West province) between districts, and was highest in districts in the North and West provinces. The geography of the two highest prevalence provinces is favourable for the occurrence of podoconiosis, given that mountains dominate in the western part of Rwanda, and volcanic chains in the north. The eastern part of the country is savanna and plains.^35^

Although most participants with podoconiosis wore footwear during the interview, many of them were wearing footwear that was not protective against exposure to soil. Additionally, many of those with lymphoedema reported that their use of footwear did not start until their early 20s, implying a substantial period of exposure to soil. Most podoconiosis cases reported living in a house with an earth or sand floor. This finding shows that key podoconiosis prevention practices (ie, consistent use of protective footwear from early age and covering floors in housing) are not widely practiced, even among individuals with the disease. Previous studies^36^ have shown that patients with podoconiosis tend to wear footwear more often than do the general population, often as a response to noticing foot swelling.

Our analysis is robust given the large sample size and widespread geographical coverage. However, a few limitations should be considered when interpreting the findings. First, the initial stage of house-to-house screening was done by community health workers who might have missed cases of early stage lymphoedema. However, the number of missed cases is probably minimal, given that 30% of all cases identified were stage one when reviewed by trained clinicians. This value is higher than the proportion reported in either Ethiopia (16·7%)^30^ or Cameroon (9·6%).^15^ Additionally, previous studies^37^ have reported the high success rate of trained community health workers in identifying podoconiosis cases.

Second, we used a small number of datapoints in our analysis. As a result, we were unable to estimate small scale spatial variation in podoconiosis prevalence. Instead, we only used long-range variation, as modelled by precipitation, elevation, enhanced vegetation index, distance from closest waterway, clay content, silt content, night light emissivity, and distance to stable night light, which has also been the case in previous work.^38^ Nonetheless, we believe that our results provide a useful operational framework for planning and setting priorities. For example, the exceedance probability map could be used to identify areas that need urgent intervention (exceedance probability around 100%) and areas where additional sampling effort is required (exceedance probability around 50%).

Lastly, we did not include data on important determinant covariates, such as footwear use and foot hygiene practice. Such data were not available in the raster surface format, which is required for such analysis. Nonetheless, we used several proximal covariates to adjust for these. Future analysis should consider the use of these covariates as they become available.

Understanding the prevalence and number of cases of podoconiosis is crucial to informing policies and setting priorities. Our investigation showed that most tropical lymphoedema cases in Rwanda are due to podoconiosis. This finding implies that the Rwanda Ministry of Health should instigate a comprehensive podoconiosis response, including interventions targeted to increase footwear and foot hygiene practice for prevention of podoconiosis. For those with the disease, ensuring access to simple hygiene-based management is critical. This access can be achieved by training health workers at health centres and district hospitals to manage cases. With expanding services, efforts must focus on community-level awareness about podoconiosis and improved patient access to services. Integration of morbidity management within general health services has been feasible in Ethiopia. Integration not only ensures sustainability but also increases access to services for people affected within reasonable reach. Rwanda is well placed to integrate these services in the national health system through the support of community health workers and primary health-care services.^28^ Inclusion of podoconiosis interventions within national health insurance will provide better financial risk protection for affected individuals and families who do not have access to appropriate interventions.
