# Supplementary material for: Geographical distribution and prevalence of podoconiosis in Rwanda: a cross-sectional country-wide survey
Source: Lancet Glob Health. 2019 Mar 27;7(5):e671–80. doi: 10.1016/S2214-109X(19)30072-5 (PMC6465958; doi:10.1016/S2214-109X(19)30072-5)
Supplement: Supplementary appendix [file mmc1.pdf]

THE LANCET
Global Health

Supplementary appendix

This appendix formed part of the original submission and has been peer reviewed. We post it as supplied by the authors.

Supplement to: Deribe K, Mbituyumuremyi A, Cano J. Geographical distribution and prevalence of podoconiosis in Rwanda: a cross-sectional country-wide survey. *Lancet Glob Health* 2019; published online March 26. http://dx.doi.org/10.1016/S2214-109X(19)30072-5.

Supplementary appendix

Geographical Distribution and Prevalence of Podoconiosis in Rwanda: A Cross-Sectional Country-wide Survey

Kebede Deribe, Aimable Mbituyumuremyi, Jorge Cano, Mbonigaba Jean Bosco BSc[3], Emanuele Giorgi, Eugene Ruberanziza, Ursin Bayisenge, Uwayezu Leonard, Jean Paul Bikorimana, Aniceth Rucogoza, Innocent Turate, Andre Rusanganwa, David M. Piggott, Rachel L. Pullan ,Abdisalan M Noor, Fikre Enquselassie, Jeanine U. Condo , Christopher J. L. Murray, Simon J. Brooker, Simon I. Hay, Melanie J. Newport, Gail Davey

Contents

1.Summary 2

2.Résumé 3

3.Sources of covariates 4

4.Model validation 7

5.Model specification 8

6.Additional results 9

7.Summary of the research protocol 11

5.1.Background and justification 11

5.2.The project background, context and need 11

5.3.Objectives 12

5.4.Stakeholders' participation 12

5.5.Definitions of the key words for this mapping survey and Literature review 12

5.6.Procedures and methods 16

5.6.1 Mapping design 16

5.6.2 *Study area* 16

5.6.3 Study population and sampling unit: 17

5.6.4 *Sample size and method:* 17

5.6.5 Data collection 18

5.6.6 Blood collection and analysis Procedures 18

5.6.7 Data management 19

5.6.8 Ethical considerations 19

5.7.Tools used in the survey 20

5.7.1 Community Form 20

5.7.2 Individual questionnaire for people with lymphoedema 21

5.7.3 Filarial FTS results 24

5.7.4 Filarial Wb123 results 24

8. References 25

1. Summary

Background: Podoconiosis is a type of tropical lymphoedema that causes massive swelling of the lower limbs. The disease is associated with both economic insecurity, due to long-term morbidity-related loss of productivity, and intense social stigma. Reliable and detailed data on the prevalence and distribution of podoconiosis are scarce. We aimed to fill this data gap by doing a nationwide community-based study to estimate the number of cases throughout Rwanda.

Methods: We did a population-based cross-sectional survey to determine the national prevalence of podoconiosis. A podoconiosis case was defined as a person with bilateral, asymmetrical lymphoedema of the lower limb present for more than 1 year, who tested negative for *Wuchereria bancrofti* antigen (determined by Filariasis Test Strip) and specific IgG4 (determined by Wb123 test), and had a history of any of the associated clinical signs and symptoms. All adults (aged ≥15 years) who resided in any of the 30 districts of Rwanda for 10 or more years were invited at the household level to participate. Participants were interviewed and given a physical examination before Filariasis Test Strip and Wb123 testing. We fitted a binomial mixed model combining the site-level podoconiosis prevalence with continuous environmental covariates to estimate prevalence at unsampled locations. We report estimates of cases by district combining our mean predicted prevalence and a contemporary gridded map of estimated population density.

Findings: Between June 12 and July 28, 2017, 1 360 612 individuals—719 730 (53%) women and 640 882 (47%) men—were screened from 80 clusters in 30 districts across Rwanda. 1143 individuals with lymphoedema were identified, of whom 914 (80%) had confirmed podoconiosis, based on the standardised diagnostic algorithm. The overall prevalence of podoconiosis was 68·5 per 100 000 people (95% CI 41·0–109·7). Podoconiosis was found to be widespread in Rwanda. District level prevalence ranged from 28·3 per 100 000 people (16·8–45·5, Nyarugenge, Kigali province) to 119·2 per 100 000 people (59·9–216·2, Nyamasheke, West province). Prevalence was highest in districts in the North and West provinces: Nyamasheke, Rusizi, Musanze Nyabihu, Nyaruguru, Burera, and Rubavu. We estimate that 6429 (95% CI 3938–10 088) people live with podoconiosis across Rwanda.

Interpretation: Despite relatively low prevalence, podoconiosis is widely distributed geographically throughout Rwanda. Many patients are likely to be undiagnosed and morbidity management is scarce. Targeted interventions through a well-co-ordinated health system response are needed to manage those affected. Our findings should inform national level planning, monitoring, and implementation of interventions.

Funding: Wellcome Trust.

2. Résumé

Distribution géographique et prévalence de la podoconiose au Rwanda: Une enquête nationale transversale.

Contexte: La podoconiose est un type de lymphœdème tropical qui provoque un gonflement massif des membres inférieurs, pouvant entraîner une stigmatisation et une dépression. Les données fiables et détaillées sur la prévalence et la distribution de la podoconiosis sont rares. Nous avons mené une étude communautaire transversale à l'échelle nationale au Rwanda pour déterminer la prévalence, la distribution géographique et le fardeau potentiel de la maladie dans tout le pays.

Méthodes: Tous les adultes (≥ 15 ans) ayant résidé dans les districts de l'étude pendant 10 ans ou plus ont été ciblés dans cette enquête transversale. Les adultes ont été recrutés au niveau des ménages. Les participants ont été interrogés, ont subi un examen physique et ont été soumis à un test de détection de l'antigène de *Wuchereria bancrofti* en circulation et de certaines IgG4 spécifiques à l'aide des tests FTS et Wb123, respectivement. Le principal indicateur de l'enquête était la prévalence des cas de podoconiose, définis comme une personne résidant dans le district de l'étude pendant au moins 10 ans et présentant un lymphœdème bilatéral asymétrique du membre inférieur présent depuis plus d'un an, qui était négative pour les tests FTS et Wb123, et avait des antécédents de signes cliniques et de symptômes associés. Nous avons ensuite ajusté un modèle binomial mixte combinant la prévalence de la podoconiose au niveau du site avec des variables environnementales continues pour estimer la prévalence de la podoconiose dans des sites non échantillonnés. Enfin, nous avons produit des estimations des cas de podoconiose par district à travers le pays, combinant notre prévalence prédite moyenne et une carte quadrillée contemporaine de la densité de population estimée.

Résultats: entre février 2013 et décembre 2017, 1, 360,612 personnes au total; 719,730 (52,9%) femmes et 640,882 (47,1%) hommes ont été examinés dans 80 grappes réparties dans 30 districts du Rwanda. Au total, 1143 personnes atteintes de lymphœdème ont été identifiées, dont 914 (79,9%) ont été confirmées comme cas de podoconiose selon un algorithme de diagnostic standardisé. La prévalence globale de la podoconiose était de 63 pour 100 000 (IC à 95%; 63 à 71). La prévalence était plus élevée chez les femmes (89 pour 100 000; IC 95%: 82-95) que chez les hommes (43 pour 100 000; IC 95%: 38-48; p <0,0001) et chez les personnes âgées de 45 ans et plus (193 pour 100 000, 178–209) que chez les personnes âgées de 15-44 ans (28 pour 100 000, 25–31; p <0,0001). La prévalence élevée de la podoconiose a été constatée dans les districts des provinces du nord et de l'ouest du Rwanda; Gicumbi, Rubavu, Burera, Musanze, Rusizi et Nyamasheke. Nos estimations suggèrent que 6,429 (3,938-10,088) personnes seraient touchées par la podoconiose au Rwanda.

Conclusions: La podoconiose est largement répandue bien que sa prévalence soit faible au Rwanda. Pourtant, de nombreux patients sont suspectés de ne pas être diagnostiqués et la gestion de la morbidité est limitée. Des interventions ciblées à travers une réponse du système de santé bien coordonnée sont nécessaires pour la provision des soins aux personnes affectées. Les résultats présentés ici sont destinés à éclairer la planification, le suivi et la mise en œuvre des interventions au niveau national.

3. Sources of covariates

Gridded continuous maps, namely raster datasets, of averaged enhanced vegetation index (EVI) for the period 2000-2015 were obtained from the African Soil Information System (AfSIS) project 1. This project generates time series average products for several environmental indicators such as vegetation indices and LST using MODIS satellite image data collected by the National Aeronautics and Space Administration (NASA). The MOD13Q1 product from MODIS library, which is updated every 16 days at 250m spatial resolution, includes vegetation indices such as Normalized Difference Vegetation Index (NDVI) and EVI [2] Day and night LST data are generated from MOD11A2 products, and have a spatial and temporal resolution of 1km and 8 days respectively [3]. Information on rainfall was extracted from a synoptic gridded map of annual precipitation calculated from monthly total precipitation gridded datasets obtained from WorldClim database [4]. This database provides a set of global climate layers obtained by interpolation of precipitation data for the period 1970–2000 collected in weather stations distributed across the world [5]. From the Consortium for Spatial Information (CGIAR-CSI), we obtained a raster dataset of elevation at $1km^2$ [6]. This elevation layer resulted from processing and resampling the gridded digital elevation models (DEM) derived from the original 30-arcsecond DEM produced by the Shuttle Radar Topography Mission (SRTM).

Soil data, including silt and clay fraction, were obtained from the ISRIC-World Soil Information project[7]. This project provides gridded maps of soil composition at 250m resolution worldwide. We also generated continuous surfaces of straight line distance (Euclidean distance) in km to the nearest water body and permanent rivers based on the Global Database of Lakes, Reservoirs and Wetlands [8] and Digital Global Chart [9], respectively.

Finally, night-light emissivity for 2013 captured by the Operational Linescan System instrument on board a satellite of the Defence Meteorological Satellite Programme was used as a proxy measure of poverty across Rwanda [10]. This instrument measures visible and infrared radiation emitted at night-time, resulting in remote imagery of lights on the ground. This information has been correlated with gross domestic product in developed countries [11] [12] and, although far from precise, can provide an indirect measure of poverty in developing countries [13].

Figure 1S. Maps of covariates used to model podoconiosis prevalence.

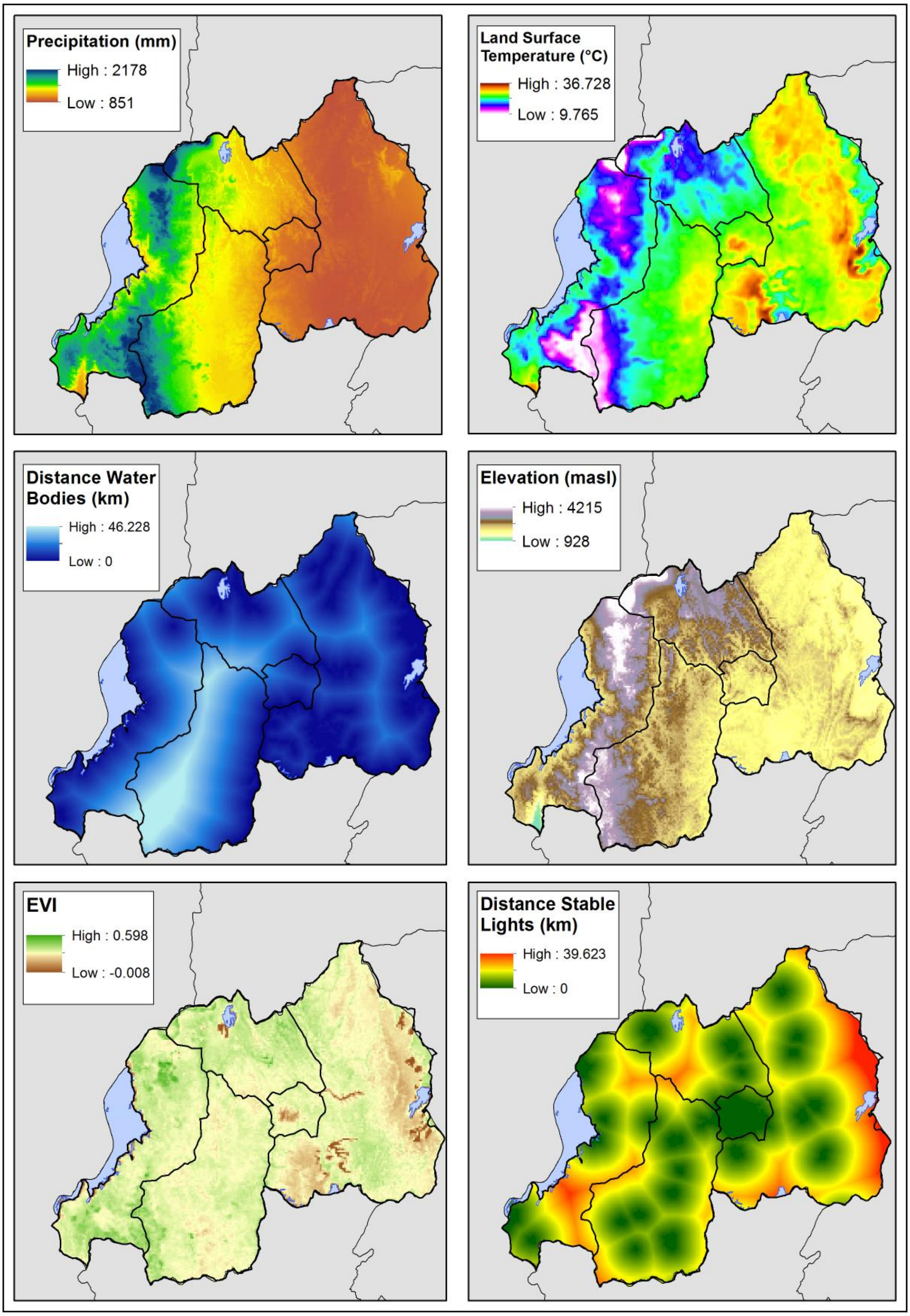

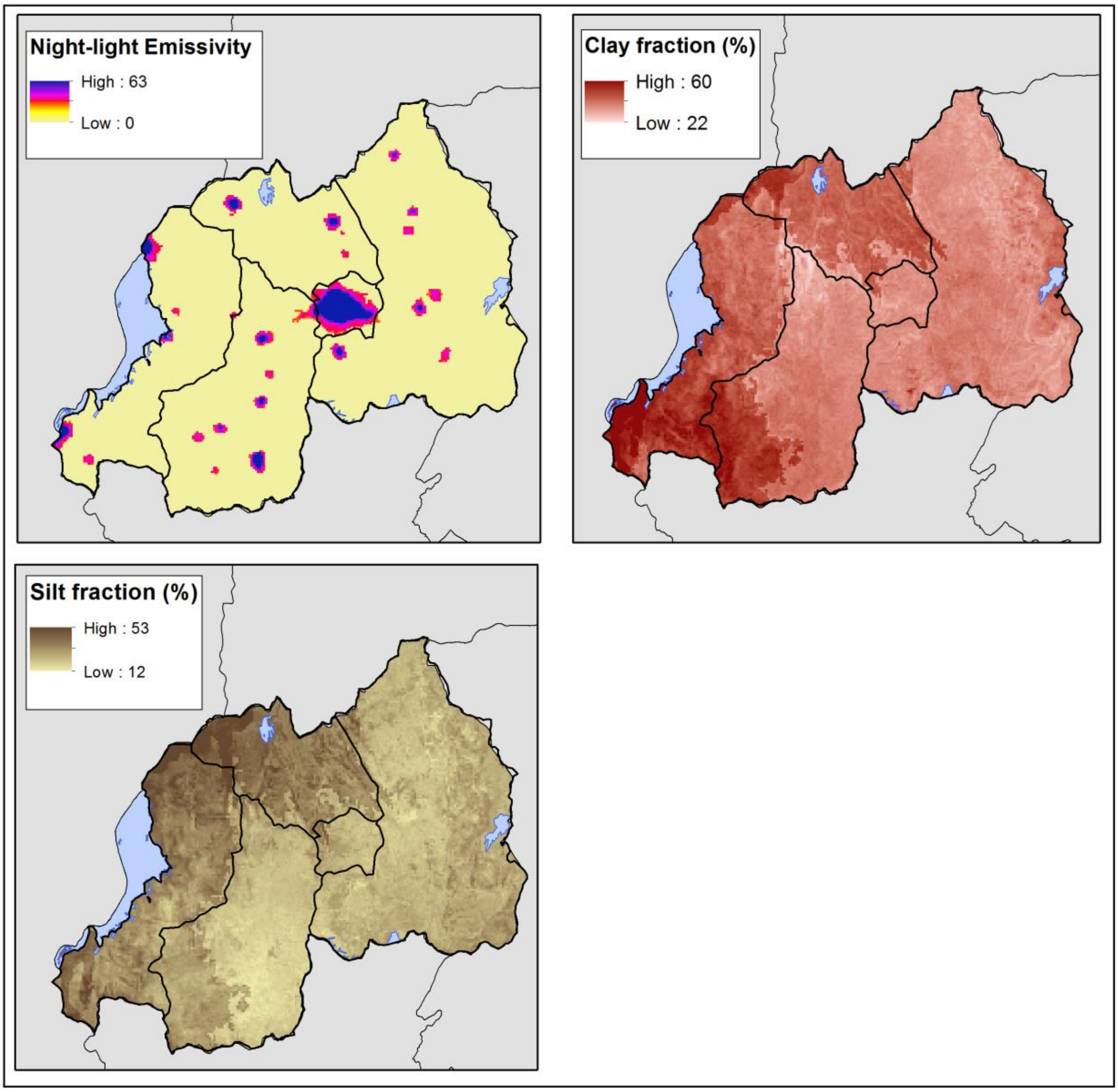
Night-light Emissivity
High : 63
Low : 0
Clay fraction (%)
High : 60
Low : 22
Silt fraction (%)
High : 53
Low : 12

4. Model validation

Figure 2S. The variogram from a non-spatial Binomial mixed model. The shaded area are the 95% confidence intervals under the assumption of independence. As the variogram falls almost entirely within this area, this implies that we do not need to fit a geostatistical model. The data do not show evidence of residual spatial correlation.

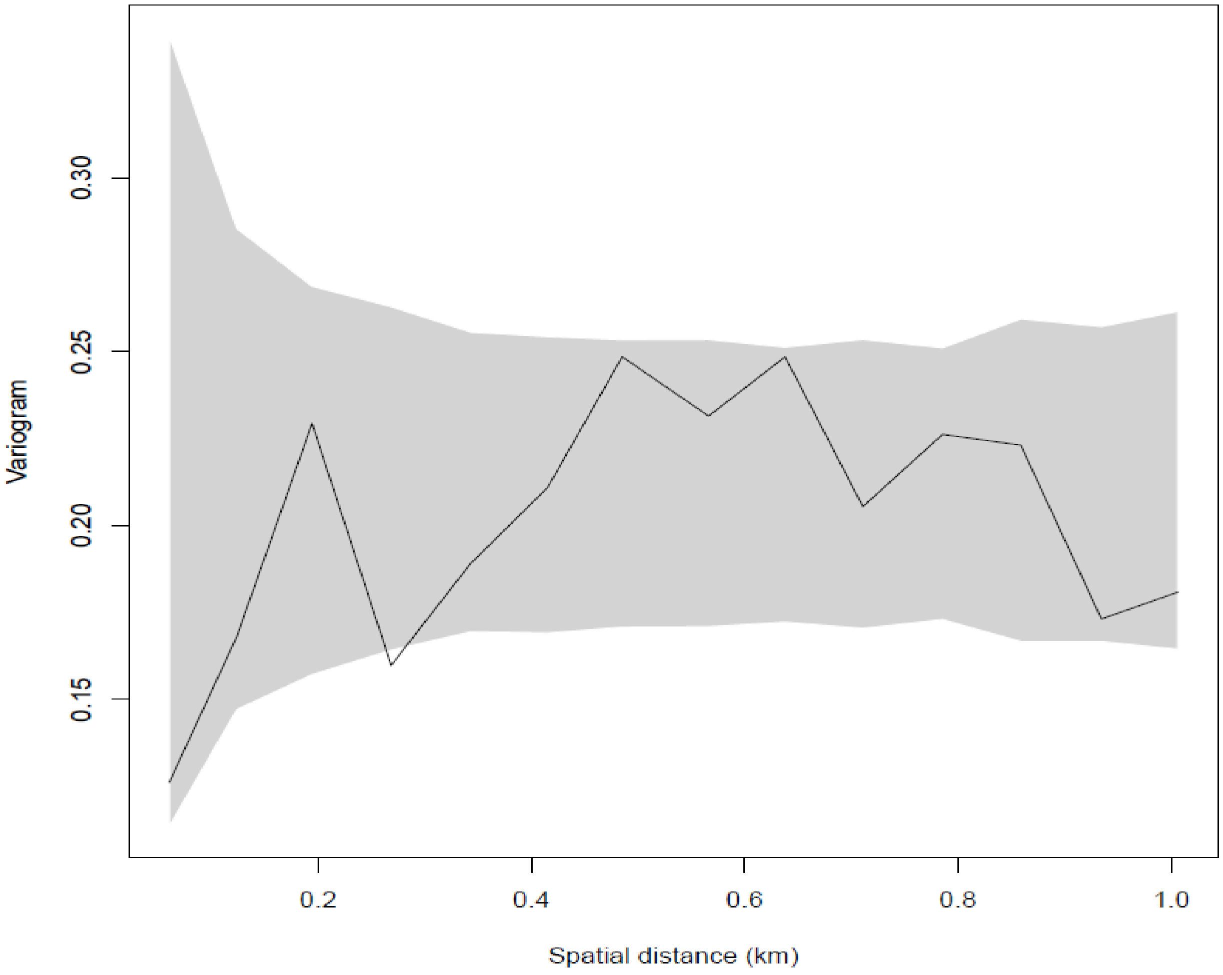

5. Model specification

Table 2: Model parameter estimates and associated 95% confidence intervals (CI).

Variable	Estimate	Lower Bound	Upper Bound
Intercept	-10.22	-11.7148	-8.7252
Precipitation	-0.0001	-0.0014	0.0012
Elevation	0.0003	-0.0006	0.0011
EVI	1.7311	-1.2691	4.7312
Slope	-0.0483	-0.1331	0.0365
Distance to closest waterway	-0.0021	-0.0202	0.0159
Silt	0.0204	-0.0153	0.0562
Clay	0.0401	0.0003	0.0799
Night Light Emission	-0.0407	-0.0671	-0.0142
Distance to stable night light	0.0132	-0.0088	0.0352

6. Additional results

Figure 3S. Predicted podoconiosis prevalence maps of Rwanda; mean predicted prevalence (A) and, lower (B) and upper 95% CI bounds (C).

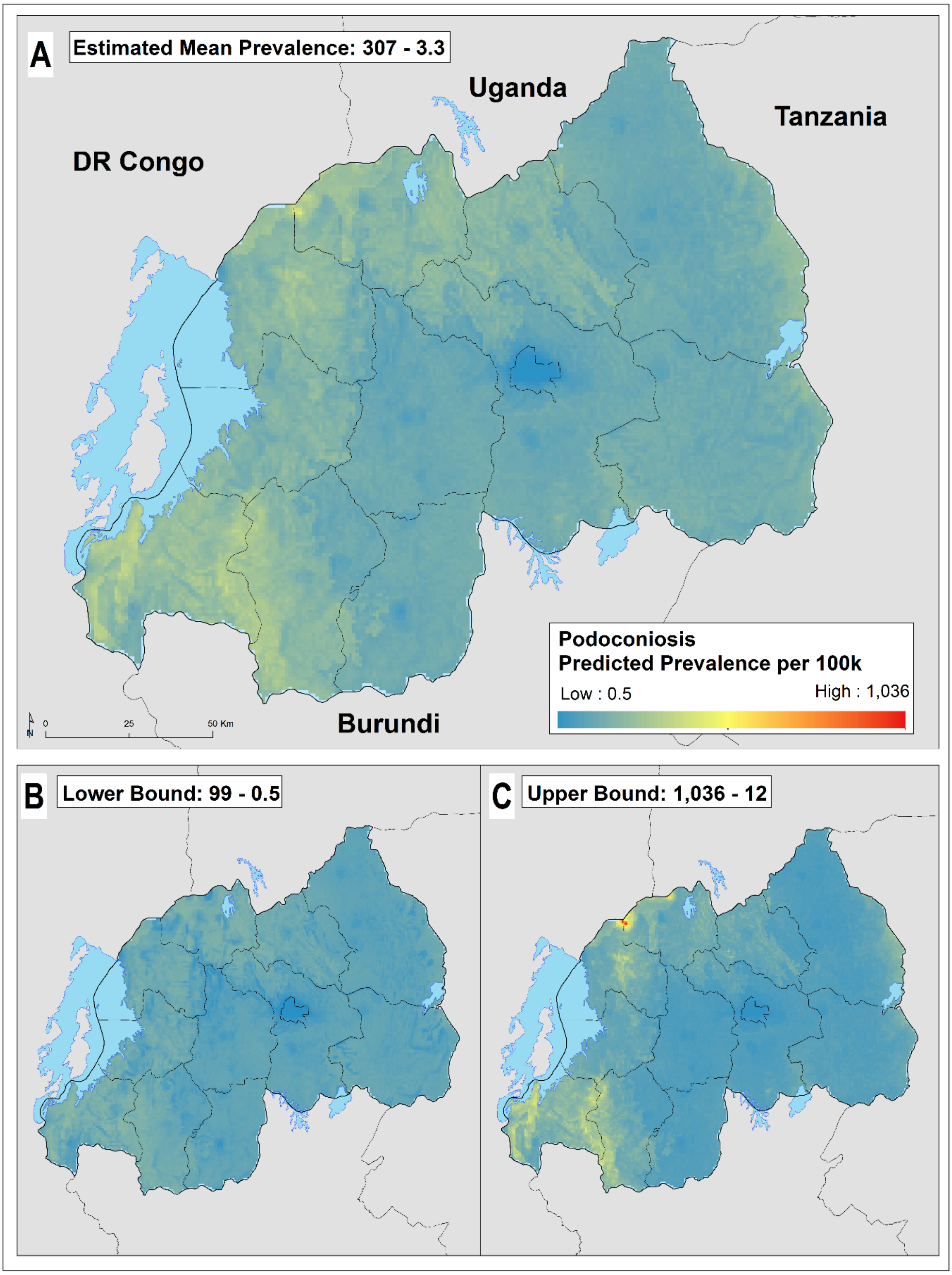

Figure 4S. Estimated number of people with podoconiosis across Rwanda: estimated number of cases (A) and, lower (B) and upper 95% CI bounds (C)

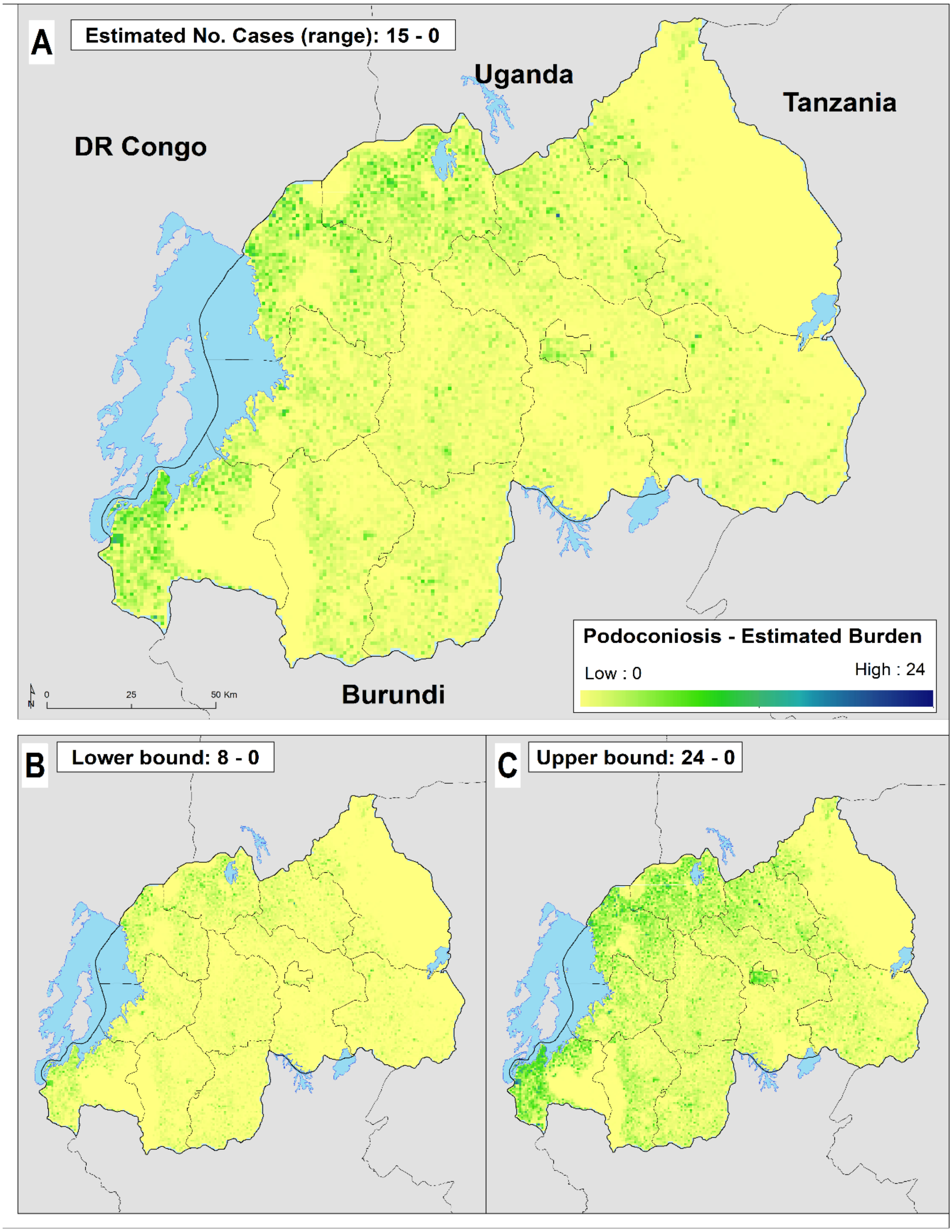

7. Summary of the research protocol

Mapping the Geographical Distribution of Podoconiosis in Rwanda

5.1. Background and justification

In 2011, podoconiosis was identified as one of the Neglected Tropical Diseases (NTDs) by the World Health Organization. Many questions remain, however, about the geographical distribution and burden of podoconiosis in many endemic countries. These include; what is the spatial distribution of podoconiosis in Rwanda and what is the burden of podoconiosis in Rwandan. This study will enable us to answer these questions. The study is designed to investigate the spatial distribution of podoconiosis, quantify the population at risk and burden of podoconiosis in Rwanda. Findings will directly inform cost-effective podoconiosis control in Rwanda and be used for future mapping of podoconiosis. Mapping distribution of podoconiosis is crucial for a number of reasons. Areas without risk of the disease need to be identified, while endemic areas need to be delineated and integrated into the control program. It is also important to define the size of the population at risk of podoconiosis in the country, both enabling effective planning and careful use of scarce resources.

5.2. The project background, context and need

Rwanda is thought to be among the countries with the highest burden of podoconiosis:[14] a non-infectious geochemical disease arising in barefoot subsistence farmers who are in long-term contact with irritant red clay soil of volcanic origins[15 16]. Due to lack of understanding of the geographical distribution of the disease, intervention against the disease is minimal.

Appropriate targeting of integrated morbidity management and disability prevention requires information on the geographical distribution and prevalence of diseases causing morbidity in order to identify high-risk areas that might benefit most from integrated control[17]. Targeting by district not only maximizes the health impact and cost-effectiveness of intervention, but also minimizes the time needed for the elimination of morbidity causing NTDs. In Rwanda, relevant information on podoconiosis prevalence and distribution is not generally available.

Previous studies have investigated the presence of lymphatic filariasis in Rwanda and concluded the absence of the disease in the country[18]. Some data are available for podoconiosis[14], but surveys to date have generally been limited to relatively small endemic areas. A recent nationwide line listing of lymphoedema cases has identified some 5,458 suspected cases all over the country. Nonetheless the causes of the lymphedema were not ascertained. Therefore, this study will be conducted among a sample of cases identified through the line listing and the causes of the lymphoedema will be established in the country.

5.3. Objectives

1. To determine the prevalence and distribution of podoconiosis in Rwanda
2. To estimate the population at risk
3. To establish which areas, qualify for control programs.

The present mapping proposal is based on international recommendations and the experience of mapping podoconiosis in Ethiopia [19-21].

5.4. Stakeholders' participation

- The RBC through the NTD &OPD Unit in MOPDD guides the overall orientation of the survey. Survey objectives and implementation strategies will be aligned with the Strategic Plan for the control of NTDs in Rwanda, 2015-2020.
- The NRL provides support for the design and implementation of the mapping. The NRL staff will guide the laboratory technicians from districts and will perform quality control.
- The Brighton & Sussex Medical School, UK is providing technical assistance to the MOH (MOPDD-RBC) for the design and also implementation of the mapping.

5.5. Definitions of the key words for this mapping survey and Literature review

Neglected Tropical Diseases (NTDs):

The Neglected Tropical Diseases (NTDs) are a group of chronic and disabling tropical infections. Currently, the World Health Organization (WHO) prioritizes 17 NTDs which affect more than 1 billion world's poorest people, and the seven most prevalent with more than 90% of the NTD global disease burden (56.6 million DALYS lost annually) are [20] - ascariasis, trichuriasis and hookworm (the three also known as soil-transmitted helminth infections), schistosomiasis, lymphatic filariasis (LF), trachoma and onchocerciasis [20].Today, the sub-Saharan Africa accounts for 99% of cases of onchocerciasis, more than 90% of the world's cases of schistosomiasis, approximately 40% of the cases of LF and trachoma, and one-third of the world's hookworm infections [20]. In 2011, podoconiosis was identified as one of the Neglected Tropical Diseases (NTDs) by the World Health Organization.

The term 'neglect' occurs at many different levels: at the community level because the NTDs arouse fear and inflict stigma at the national level because the NTDs occur in remote and rural areas and are often a low priority for health ministers; and at the international level because they are not perceived as global health threats equivalent to the high-mortality big three conditions - HIV/AIDS, Malaria and Tuberculosis. Podoconiosis is among the first NTDs which inflict stigma.

Podoconiosis:

Podoconiosis or endemic non-filarial elephantiasis is a non-infectious geochemical type of disease caused by exposure of bare feet to red clay soil derived from volcanic rocks (Price EW 1976). Although the condition has been known for more than a millennium, it has been neglected and less researched. Of recent, podoconiosis has been classified as neglected tropical disease by World Health Organization (www.podo.org)
The disease occurs in highland red clay soil areas, mainly among poor, bare footed agricultural communities, who do not wear protective shoes and/or wash the dust off their feet using soap and water.

Podoconiosis is a non-filarial, non-infectious lymphoedema of the lower limb. The disease is geochemical and caused by exposure to red clay soil [16]. The disease is a complex interaction between gene and environment occurring over many years. Mineral particles from the soil penetrate the skin and are taken up by macrophages in the lymphatic system which causes inflammation and fibrosis of the vessel lumen leading to blockage of the lymphatic drainage. This results in oedematous feet and legs and subsequently progresses to elephantiasis and nodular skin changes [22]. The disease causes progressive bilateral swelling of the legs [23].

To achieve rapid and community wide impact, the scale-up of interventions is very important. One of the critical impediments for the scaling up of interventions is that the distribution of podoconiosis in space and time is not clearly understood. The absence of a global strategy may partly be attributed to the lack of clear understanding of the geographical distribution of the disease. Most countries are considered endemic based on historical and anecdotal reports. Lack of clinical suspicion and diagnostic tests has probably underestimated podoconiosis prevalence in the past. The disease is not well-known by health workers and within the health system and is often confused with lymphatic filariasis, another major cause of lymphoedema in the tropics. There were a few mapping attempts documented in some endemic countries, but these data are as old as 40 years. Recent localized studies have also been conducted, but these are limited to a few endemic areas. Risk maps based on climate and environmental measures linked with data from past surveys can broadly predict where occurrence is possible, but not enough reliable historical data exists.
Earlier studies identified variation in genetic susceptibility at individual and household levels, which resulted in clustering of the disease among susceptible families [24-26]. Risk differences among individuals, households and communities might not be confined only to genetic susceptibility and may relate to individual protective and preventive behaviours, while the large-scale differences may rely upon differences in the environment. Only a few studies have identified risk factors at individual and community levels [27 28].

Podoconiosis affects low-socioeconomic, genetically susceptible individuals who often go barefoot [16 22]. It causes gross bilateral, often below-the-knee lymphoedema of the lower limb. The term podoconiosis was coined from two Greek words *podos* and *konos,* which mean foot and dust, respectively, and implies that the disease is caused by exposure of feet to irritant clay soil [22 29 30].

Podoconiosis is a multifactorial disease with evidence of genetic susceptibility and environmental exposure. The pathogenesis of podoconiosis is not clearly understood. Based on existing evidence, the widely accepted cause is that mineral particles induce inflammation among individuals who are genetically susceptible to the disease. This interaction of genetic factors and the environment will induce an inflammatory reaction which leads to fibrosis of the lymphatic vessel lumen. Although the mechanism is yet to be studied, it is hypothesised that the mineral particles are engulfed by macrophages and induce an inflammatory response in the vessels which leads to fibrosis, oedema and gross elephantiasis [22].

The disease only affects some of the barefooted individuals -not all exposed individuals develop podoconiosis. In his clinic during the 1970s in Addis Ababa, Ethiopia, Price noticed that affected individuals would often bring their affected relatives or report that they had affected relatives. Subsequently, he studied 90 families and concluded that the existence of autosomal recessive traits was a factor in the disease. The gene frequency was calculated to be 30% (range 15-40%) in the general population [25].

A 2005 pedigree study among 59 multi-generational families with multiple affected members in southern Ethiopia presented evidence for a genetic basis to podoconiosis. The study illustrated that both genetic and environmental factors contribute to the pathophysiology of podoconiosis [24]. A genome-wide comparison of the frequency of genetic variants between podoconiosis cases and unaffected controls from southern Ethiopia revealed that genetic variants in the HLA locus (a genomic region on chromosome 6) confer susceptibility to podoconiosis. Each of the known susceptibility alleles confers an increased podoconiosis risk, with odds ratios of between 1·79 and 2.0. The location of the risk alleles within the HLA locus suggests that podoconiosis is a T-cell mediated inflammatory condition [26].

Environment plays important role in the causation of podoconiosis, the type of soil and soil characterises dictated by climate and environmental factors generate and give the appropriate mineral content and physical properties for the putative causes. Biological factors such as genetic susceptibility, age and sex determines individual’s risk of acquiring the disease. Behavioural factors such as footwear use and foot hygiene determine the exposure of individuals to irritant soil particles. Contextual factors such as household economy and access to water determines how individuals practice protective behaviours such as footwear use and foot hygiene.

Epidemiological and observational studies have indicated some environmental factors to be associated with podoconiosis. Price noted an association of red clay soil with podoconiosis. In his study, he noted a rapid fall of podoconiosis prevalence outside red soil areas. The prevalence of podoconiosis decreased from 6.92% to 2.96% at the edge of the red soil, and further decreased to 0.79% and 0.98%, 25 kilometres away from the edge in two different directions [31]. Climatic factors which may affect the type of soil generated were found to be associated with the high prevalence of podoconiosis. In earlier studies, it was postulated that areas with high prevalence of podoconiosis are characterized by red clay soil, altitude> 1500 masl, and annual rain fall of 1000mm [31].

The exact cause of podoconiosis in red clay soil is yet to be studied. Previous studies indicated a range of mineral particles. Price suggested that silicate particles cause subendothelial oedema, endolymphangitis, collagenisation and obliteration of the lymphatic lumen [32]. Overlaying the prevalence of podoconiosis on geological maps indicated that there was some correlation between alkali basalt rocks and podoconiosis [33]. The weathering of such rocks produces reddish soil with fine size which is capable of penetrating intact skin. Soil in the highland endemic areas are more likely to be characterized by particle size <5µm size than those found in non-endemic lowland areas [34]. Fyfe and Price [35] injected silica into the lymphatics of the lower extremities of rabbits and found similar changes with that of podoconiosis. Frommel and colleagues [36] also suggested that beryllium and zirconium might be the cause of podoconiosis. A recent study involving geological, spatial, and soil chemical analyses showed that smectite, mica and quartz within the soil were associated with podoconiosis [28]. Some of these particles have been documented to induce an inflammatory response and fibrosis.

Podoconiosis is common among barefoot subsistence farmers, who are exposed to red clay soil due to their work. The onset of the disease is more common in the second and third decades of life with the disease occurring up to the sixth decade [24]. The prevalence in the first decade is almost zero. A study from Uganda reported no cases among <10 years old [37], and in southern Ethiopia only 6.8% of the patients were ≤15 years old [37 38]. There are inconsistent findings about the sex ratio in podoconiosis distribution. Most studies indicated that females are disproportionately affected than male [39-42], while one study indicated that male are more affected than female[43]. Few studies found out that no sex difference in the distribution of the disease[37 38]

5.6. Procedures and methods

5.6.1 Mapping design

Population-based prevalence surveys (PBPS) are the gold standard methodology for obtaining accurate disease estimates when case detection and reporting through the health system is incomplete, and these have been used to provide sub-national estimates of disease distributions for many diseases. For less common outcomes (fewer than 1 case in 1000 individuals) however, standard PBPS rapidly become unfeasible. Given that the expected prevalence range for podoconiosis in endemic regions is low, the PBPS approach requires adaptation to achieve the sample sizes needed to generate sufficiently precise prevalence estimates.

One alternative to randomly sampling individuals or households is to screen all residents within each sampling cluster. House-to-house screening by mobile expert teams would likely yield the highest number of cases, but such a strategy would be expensive and difficult to sustain. As an alternative, trained village volunteers have been used during programmatic activities to effectively detect and refer people with lymphoedema. Given how difficult it is to diagnose podoconiosis accurately, using community volunteers to perform an exhaustive house-to-house case search would require follow up expert case validation. The success of such an approach would thus rely on well-trained village volunteers being able to recognise possible conditions, and a highly skilled, mobile case-validation team to confirm all potential cases.

The purpose of this mapping protocol is to determine national and district prevalence and geographic distribution of podoconiosis in Rwanda. A three-step training, screening and case verification process will be conducted at randomly selected clusters (referred to sectors in this protocol) in suspected districts across the country to generate nationally, and district prevalence of podoconiosis. To distinguish podoconiosis cases from other causes of lymphoedema, a clinical algorithm will be used [20].

5.6.2 Study area

All 30 districts of Rwanda will be included in the study. Rwanda is divided into 30 districts and all have reported suspected cases of lymphoedema.

5.6.3 Study population and sampling unit:

All 30 suspected districts will be mapped. These are referred to throughout the protocol as districts. For each district, the survey is powered to generate prevalence estimates for podoconiosis at a precision of 0.003%, assuming a conservative prevalence estimate of 4.2 case per 10,000. The survey will use a cluster sampling design.

5.6.4 Sample size and method:

Sample size calculations are based on an expected prevalence of 4.2 per 10,000, reflecting conservative estimates for the prevalence of lymphedema.

Using standard sample size formula:

$$n = \frac{DEFF.z^2.P(1-P)}{e^2.R}$$

Where:
DEFF = the design effect
z = standard normal deviate corresponding to 95% confidence intervals
P = expected prevalence
R = community participation rate
e = precision
and a finite population correction factor of:

$$n_{adj} = \frac{n.N}{n+(N-1)}$$

Where:
N = total population size of the district

Assuming a design effect of 1.6 (a design effect of 1.6 was calculated from a mapping survey in Cameroon) and a community participation rate of 80%, below shows the precision when targeting for screening: With finite population correction 32,879 individuals per district were estimated. Overall we estimated a sample size of 986,376 individuals to be screened from 60 sectors (two sectors per district) nationally. To adjust for sector size, sectors were assigned to districts that were proportional to the size of sectors per district. The adjustment resulted in 80 of 416 sectors being selected, with independent selection in each district.

Community volunteers will conducted active community-based case finding, screening all members of a community for swelling of the lower leg. All self-reported cases will be documented as suspected cases during an exhaustive house-to-house census and case listing. Community volunteers will be provided case definitions to identify the cases.

5.6.5 Data collection

Expert clinical diagnostic teams will verify all suspected cases of lymphedema (listed by CHWs) in the selected sectors. Each team will comprise three health workers, two nurse with previous experience of diagnosing podoconiosis in the routine health system and a laboratory technician. All data will be collected using the LINKS software package on Android smartphones[44]. Individual data will be collected on age, sex, education, occupation, place of residence, shoe wearing, foot hygiene practices. Household data on Water, Sanitation and Hygiene (WASH) variables, including source of water will be recorded. A simple checklist will be used to examine for possible differential diagnoses of podoconiosis. In those clinically confirmed cases to have podoconiosis, duration of illness, shoe wearing practice and disease stage will be recorded. In addition, information from physical examination including preservation of sensation in the toes, clinical signs of leprosy or onchocerciasis, and groin involvement will be recorded.

All lymphedema cases in a selected cluster will be screened for filariasis using Alere Filariasis Test Strip tests and wb123 which will be performed by trained laboratory technicians, according to the manufacturer's instructions[45],[46]. Community GPS coordinates will be collected using smartphones.

5.6.6 Blood collection and analysis Procedures

After obtaining informed consent from the participant, the test will be performed by trained laboratory technicians, according to the manufacturer's instructions. The patient's third or fourth finger will be cleaned with 70% alcohol and punctured using a sterile lancet. The initial sample of blood will be removed using a cotton swab, and sufficient fresh blood will be obtained to fill a 75-μl capillary tube. The blood will be transferred from the capillary tube to the pad on a Filariasis Test Strip (FTS) card. The result of each FTS card will be read at 10 minutes exactly, not before, and not after. A positive result shows two lines, and a negative result will show a single line. The test will be repeated if the control line is not shown. Test results with the individual's ID number will be recorded both on the card, and on each individual's data sheet.

In addition to the FTS test, Wb123 test will be performed. This test will also be performed by trained laboratory technicians, according to the manufacturer's instructions. The patient's third or fourth finger will be cleaned with 70% alcohol and punctured using a sterile lancet. The initial sample of blood will be removed using a cotton swab, and sufficient fresh blood will be obtained to fill a 10-μl capillary tube. The blood will be transferred from the capillary tube to the pad on a WB123 card drawn to back line and dispense 4 drops of assay diluent vertically into the square assay diluent well. The result of each FTS card will be read at 30 minutes. A

positive result shows two lines, and a negative result will show a single line. The test will be repeated if the control line is not shown. Test results with the individual's ID number will be recorded both on the card, and on each individual's data sheet.

5.6.7 Data management

Each data collection team will be given a unique number, and data quality will be monitored online by two supervisors with access to the database. The supervisors will check daily on data quality and give feedback to each data collection team. If there are inconsistencies, the supervisor will discuss with the team leaders to correct them.

5.6.8 Ethical considerations

Ethical approval will be obtained from Rwanda National Ethics Committee and from the UK. Study participants will have the purpose of the study explained to them in their local language at the time of recruitment. Confidentiality will be maintained using ID codes. Names of participants will not be recorded. Individual informed written consent will be obtained from each participant (≥18 years of age). Additionally, for those 15 to 18 years old, consent will be obtained from their parents/guardian and the participant themselves will provide informed assent. Patient information sheets and assent and consent forms will be in French and English languages, but as the need arises, these forms will be translated to the local language of the specific population and/or ethnic group. As part of the podoconiosis elimination programs, Rwanda will put in place a morbidity management and disability prevention plan that will include provision of care for the patients suffering from lymphoedema. People with podoconiosis with depression will be referred to the nearby health facility with psychiatric services. This mapping will use approaches that are invasive (i.e., blood draws).

5.7. Tools used in the survey

5.7.1 Community Form

Date	*(DD-MMM-YYYY)*	|__|__|-|__|__|__|-|__|__|__|__|	Record Taker's Initials	|__|__|__|

Community form			
**SN**	**Questions and Filters**	**Response & Coding Categories**	**Skip**
101	District name		
102	Sector name		
103	Health center name		
104	Click the 'Record Location' button on the screen of the phone and wait for the GPS coordinates to be recorded.		
105	Total sector population		
106	Has this community received deworming treatment last year? (April/2016)	☐1 = Yes<br>☐2 = No	
107	Has this community received deworming treatment this year? (March or October/2017)	☐1 = Yes<br>☐2 = No	
107	Is this community urban or rural?	☐1 = Rural<br>☐2 = Urban	

5.7.2 Individual questionnaire for people with lymphoedema

Date	(*DD-MMM-YYYY)*	|__|__|-|__|__|__|-|__|__|__|__|	Record Taker's Initials	|__|__|__|

Section 1 Demographic and Socioeconomic Information			
**SN**	**Questions and Filters**	**Response & Coding Categories**	**Skip**
101	Does the person have lymphoedema?	☐ 0 = No →<br>☐ 1 = Yes (verify by observation)	End
102	Was the participant identified in previous registration by CHWs? (Refer the list)	☐ 1 = Yes ☐ 2 = No	
103	Read the barcode for consent		
104	District name		
105	Sector name		
106	Health Centre Name		
107	Individual ID (DD/SSS/Part.)		
108	Sex Check box (✓)	☐ 1 = Male ☐ 2 = Female	
109	How old are you?	Age (In years at last birthday >=15)	
110	Religion	☐ 1 = Muslim☐ 2 = Christian ☐ 3 = Other	
111	How long you lived in the current location?	Years	
112	What is your major occupation currently? (Whatever you do to earn money)?	☐ 1 = Employed (Government or private agencies)<br>☐ 2 = Businessman/women<br>☐ 3 = Farmer<br>☐ 4 = Housewife<br>☐ 5 = Daily labourer<br>☐ 6 = Student<br>☐ 7 = Have no Job<br>☐ 8 = Retired<br>☐9 = Other specify ____________	
113	Are you able to read and write in any language?	☐ 1 = Yes ☐ 2 = No	
114	Grade completed	☐ 0 = No formal education<br>☐ 1 = Primary<br>☐ 2 = Secondary<br>☐ 3 = Tertiary	
115	What is your current marital status? Check box (✓)	☐1=Single ☐2= Married<br>☐3=Divorced ☐4 = Widowed	
**Section 2 Household questions**			
201	What type of floor does your house have?	☐1 = Earth/sand<br>☐2 = Dung<br>☐3 = Wood/planks<br>☐4 = Palm/bamboo<br>☐5 = Parquet or polished<br>☐6 = Wood<br>☐7 = Vinyl or asphalt strips<br>☐8 = Ceramic tiles<br>☐9 = Cement<br>☐10 = Carpet<br>☐11 = Other	
202	Source of drinking water?	☐1 = Pipe-borne<br>☐2 = River/stream<br>☐3 = Borehole/well<br>☐4 = Pond/stagnant	
203	Where is that water source located?	☐1 = In own dwelling<br>☐2 = In own yard/plot	Q107

		☐3 = Elsewhere	
204	How long (mins) does it take to go there, get water, and come back?		Q108
**Section 3 Shoe wearing and foot care practice**			
301	Have you ever worn shoes?	☐ 1 = Yes<br>☐ 2 = No →	Go to Q206
302	How old were you when you first got shoes?		
303	Is the person wearing shoes at the time of the interview?	☐ 1 = Yes<br>☐ 2 = No →	Go to Q205
304	Describe the shoes the person is wearing.	☐1= Hard plastic ☐2= Open sandal<br>☐ 3=Leather ☐ 4=Canvas ☐ 5=other	
305	When do you wear shoes? (multiple answers possible)	☐1= At home<br>☐ 2=During rainy season<br>☐3= On market days<br>☐4= On the field<br>☐5= On Sundays<br>☐ 6=When walking far	
306	When do you wash your feet?	☐1=Whenever they are dirty<br>☐2=Before sleeping<br>☐3=Before prayer<br>☐9=Other (specify)________	
307	How often do you wash your feet very carefully so that they are very clean?	☐1= More often than once a day<br>☐2= Daily<br>☐ 3=Less often than daily, but more often than weekly<br>☐4= Weekly or less often	
**Section 4 Leg swelling history and physical examination**			
402	Do you have any family member (living or dead) with history of leg swelling?	☐ 0 = No →<br>☐ 1 = Yes	Go to Q 304
403	How many people in your family (living or dead) have leg swelling?		
404	How old were you when you first noticed this swollen leg?		
405	Where did the swelling start from?	☐1= From high up<br>☐2= From the foot or lower leg	
406	Do you have history of rheumatic heart disease?	☐ 0 = No ☐ 1 = Yes	
407	Do you have swelling in the groin area?	☐ 0 = No ☐ 1 = Yes	
408	Are you diagnosed as a leprosy patient?	☐ 0 = No ☐ 1 = Yes	
409	Is there preservation of sensation in the toes? (Physical examination)	☐ 0 = No ☐ 1 = Yes	
410	Does the person have any signs and symptoms of onchocerciasis?	☐ 0 = No ☐ 1 = Yes	
411	Did the swelling start after a major surgical procedure?	☐ 0 = No ☐ 1 = Yes	
412	Did the swelling start at birth?	☐ 0 = No ☐ 1 = Yes	
413	Is the swelling present in both legs?	☐ 1 = Both legs<br>☐ 2 = One leg →	Go to Q415
414	If in both legs, does the size of the swelling differ between the two legs?	☐ 0 = No<br>☐ 1 = Yes	
415	Does the person have swelling in other part of the body?	☐ 0 = No →<br>☐ 1 = Yes	Go to Q417
416	If yes to Q415which part of the body? (Multiple answers possible)	☐ 1= Upper limb<br>☐ 2= Breast<br>☐ 3= Vulva/Penis<br>☐ 4= Hydrocele<br>☐ 5=Other: Specify:	
417	Does the person have Chyluria (Milky Urine)?	☐ 0 = No	

		☐ 1 = Yes	
418	Did the person experience acute attack in the past 6 months?	☐ 0 = No<br>☐ 1 = Yes	
419	Podoconiosis disease stage	☐ 1 = Stage 1 ☐ 2 = Stage 2<br>☐ 3 = Stage 3 ☐ 4 = Stage 4<br>☐ 5 = Stage 5	

**Section 5 PHQ-9 for people with lymphoedema**	**Code**

**Section 6. Medication history and Knowledge:** Have you ever seek or received:			
601	Deworming medicines during campaign (March/October) this year?	Yes	1
		No	0
		Don’t know	99
602	Deworming medicines during campaign (April 2016)?	Yes	1
		No	0
		Don’t know	99
603	Traditional medicines for this swelling (lymphoedema)?	Yes	1
		No	0
		Don’t know	99
604	Modern medicines (HFs or Pharmacy) for this swelling (lymphoedema)?	Yes	1
		No	0
		Don’t know	99
605	Do you know how to get this condition/disease?	Yes	1
		No	0

The end!

Thank you for giving us your time and answers to many questions. We hope this will help the work in the future.

5.7.3 Filarial FTS results

Read the barcode for test results	
Lab Tech initials	
Record taker's initials	
FTS Results	1. Negative<br>2. Positive<br>3. Indeterminate

5.7.4 Filarial Wb123 results

Read the barcode for test results	
Lab Tech initials	
Record taker's initials	
Wb123 test Results	1. Negative<br>2. Positive<br>3. Indeterminate

8. References

1. Africa Soil Information System. Available at http://www.africasoils.net/data/datasets Accessed on January 20, 2014.

2. DAAC NL. NASA LP DAAC: MOD13Q1 Vegetation Indices 16-Day L3 Global 250m: NASA EOSDIS Land Processes DAAC, USGS Earth Resources Observation and Science (EROS) Center, Sioux Falls, South Dakota (https://lpdaac.usgs.gov); [Available from: https://lpdaac.usgs.gov accessed January 2017.

3. DAAC NL. NASA LP DAAC: MOD11A2 Land Surface Temperature and Emissivity 8-Day L3 Global 1km: NASA EOSDIS Land Processes DAAC, USGS Earth Resources Observation and Science (EROS) Center, Sioux Falls, South Dakota (https://lpdaac.usgs.gov); [Available from: https://lpdaac.usgs.gov accessed January 2017.

4. WorldClim. Global Climate data [Available from: http://www.worldclim.org/.

5. Fick Stephen E, Hijmans Robert J. WorldClim 2: new 1-km spatial resolution climate surfaces for global land areas. *International Journal of Climatology* 2017;37(12):4302-15. doi: 10.1002/joc.5086

6. CGIAR-CSI. Consortium for Spatial Information [Available from: http://www.cgiar-csi.org/.

7. ISRIC - World Soil Information. Soil property maps of Africa at 1 km. Available for download at www.isric.org. Accessed on 20 Jan 2014.

8. Lehner B, Döll P. Development and validation of a global database of lakes, reservoirs and wetlands. *Journal of Hydrology* 2004;296(1–4):1-22. doi: https://doi.org/10.1016/j.jhydrol.2004.03.028

9. DIVA-GIS. Digital Global Chart: inland waters [Available from: http://www.diva-gis.org/gdata.

10. Elvidge CD, Baugh KE, Kihn EA, et al. Mapping city lights with nighttime data from the DMSP operational linescan system. *Photogrammetric Engineering and Remote Sensing* 1997;63(6):727-34.

11. Doll CNH, Muller JP, Morley JG. Mapping regional economic activity from night-time light satellite imagery. *Ecological Economics* 2006;57(1):75-92.

12. Ebener S, Murray C, Tandon A, et al. From wealth to health: modelling the distribution of income per capita at the sub-national level using night-time light imagery. *Int J Health Geogr* 2005;4(1):5. doi: 10.1186/1476-072X-4-5

13. Noor AM, Alegana VA, Gething PW, et al. Using remotely sensed night-time light as a proxy for poverty in Africa. *Popul Health Metr* 2008;6:5. doi: 10.1186/1478-7954-6-5 [published Online First: 2008/10/23]

14. Price EW. Endemic elephantiasis of the lower legs in Rwanda and Burundi. *Trop Geogr Med* 1976;28 283-90.

15. Davey G, Newport M. Podoconiosis: the most neglected tropical disease? . *Lancet* 2007;369:888-9.

16. Davey G, Tekola F, Newport MJ. Podoconiosis: non-infectious geochemical elephantiasis. *Trans R Soc Trop Med Hyg* 2007 101(12):1175-80.

17. World Health Organization. Lymphatic Filariasis Managing Morbidity And Preventing Disability. World Health Organization. Geneva, Switzerland. 2013

18. Ruberanziza E, Mupfasoni D, Karibushi B, et al. Mapping of Lymphatic Filariasis In Rwanda. *Journal of Lymphoedema* 2009;4(1):20-23.

19. Deribe K, Brooker SJ, Pullan RL, et al. Epidemiology and individual, household and geographical risk factors of podoconiosis in Ethiopia: results from the first nationwide mapping. *Am J Trop Med Hyg* 2014:148–58.

20. Sime H, Deribe K, Assefa A, et al. Integrated mapping of lymphatic filariasis and podoconiosis: lessons learnt from Ethiopia. *Parasit Vectors* 2014;7(1):397.

21. WHO. Operational guidelines for rapid mapping of Bancroftianfilariasis in Africa. Geneva: . *World Health Organization;WHO/CDS/CPE/CEE/20009* 2000

22. Price E. Podoconiosis:Non-filarial Elephantiasis. *Oxford Medical Publications, Oxford* 1990

23. WHO. Neglected tropical diseases: Podoconiosis: endemic non-filarial elephantiasis. Accessed on 16 May ; Available at [http://www.who.int/neglected_diseases/diseases/podoconiosis/en/] 2012 [

24. Davey G, GebreHanna E, Adeyemo A, et al. Podoconiosis: a tropical model for gene-environment interactions? . *Trans R Soc Trop Med Hyg* 2007;101(1):91-6.

25. Price EW. A possible genetic factor in non-filarial elephantiasis of the lower legs. *Ethiop Med J* 1972;10(3):87-93.

26. Tekola Ayele F, Adeyemo A, Finan C, et al. HLA class II locus and susceptibility to podoconiosis. *N Engl J Med* 2012;366(13):1200-08.

27. Molla YB, Le Blond JS, Wardrop N, et al. Individual correlates of podoconiosis in areas of varying endemicity: a case-control study. *PLoS Negl Trop Dis* 2013;7(12):e2554.

28. Molla YB, Wardrop NA, Le Blond JS, et al. Modelling environmental factors correlated with podoconiosis. *Int J Health Geogr* 2014;13(1):24.

29. Price EW. Non-Filarial Elephantiasis - Confirmed as a Geochemical Disease, and Renamed Podoconiosis. *Ethiop Med J* 1988;26(3):151-53.

30. Tomczyk S, Deribe D, Brooker SJ, et al. Association between Footwear Use and Neglected Tropical Diseases: A Systematic Review and Meta-Analysis. *PLoS Negl Trop Dis* 2014;8(11):e3285.

31. Price EW. The relationship between endemic elephantiasis of the lower legs and the local soils and climate *Trop Geogr Med* 1974;26(3):225-30.

32. Price E. The association of endemic elephantiasis of the lower legs in East Africa with soil derived from volcanic rocks. *Trans R Soc Trop Med Hyg* 1976;4:288- 95.

33. Price EW. The association of endemic elephantiasis of the lower legs in East Africa with soil derived from volcanic rocks. *Trans R Soc Trop Med Hyg* 1976;70(4):288-95.

34. Price EW, Plant DA. The significance of particle size of soils as a risk factor in the etiology of podoconiosis. *Trans R Soc Trop Med Hyg* 1990;84(6):885-6.

35. Fyfe NC, Price EW. The effects of silica on lymph nodes and vessels--a possible mechanism in the pathogenesis of non-filarial endemic elephantiasis. *Trans R Soc Trop Med Hyg* 1985;79(5):645-51.

36. Frommel D, Ayranci B, Pfeifer HR, et al. Podoconiosis in the Ethiopian Rift Valley. Role of beryllium and zirconium. *Trop Geogr Med* 1993;45(4):165-67.

37. Onapa AW, Simonsen PE, Pedersen EM. Non-filarial elephantiasis in the Mt. Elgon area (Kapchorwa District)of Uganda. *Acta Trop* 2001;78(2):171-6.

38. Desta K, Ashine M, Davey G. Prevalence of podoconiosis (endemic non-filarial elephantiasis) in Wolaitta, Southern Ethiopia. *Tropical Doctor* 2003;33(4):217-20.

39. Alemu G, Tekola Ayele F, Daniel T, et al. Burden of podoconiosis in poor rural communities in Gulliso woreda, West Ethiopia. *PLoS Negl Trop Dis* 2011;5(6):e1184.

40. Molla YB, Tomczyk S, Amberbir T, et al. Podoconiosis in East and West Gojam zones, Northern Ethiopia. *PLoS Negl Trop Dis* 2012;6(7):e1744.

41. Tekola Ayele F, Alemu G, Davey G, et al. Community-based survey of podoconiosis in Bedele Zuria woreda, southwest Ethiopia. *Int Health* 2013; 5(2):119-25.

42. Wanji S, Tendongfor N, Esum M, et al. Elephantiasis of non-filarial origin (podoconiosis) in the highlands of north-western Cameroon. *Ann Trop Med Parasitol* 2008;102(6):529-40.

43. Geshere Oli G, Tekola Ayele F, Petros B. Parasitological, serological, and clinical evidence for high prevalence of podoconiosis (non-filarial elephantiasis) in Midakegn district, central Ethiopia. *Trop Med Int Health* 2012;17(6):722-26. doi: 10.1111/j.1365-3156.2012.02978.

44. Pavluck A, Chu B, Flueckiger RM, et al. Electronic Data Capture Tools for Global Health Programs: Evolution of LINKS, an Android-, Web-Based System. *PLoS Negl Trop Dis* 2014;8(4):e2654.

45. Weil GJ, Curtis KC, Fakoli L, et al. Laboratory and field evaluation of a new rapid test for detecting Wuchereria bancrofti antigen in human blood. *Am J Trop Med Hyg* 2013;89(1):11-15.

46. Rao RU, Atkinson LJ, Ramzy RM, et al. A real-time PCR-based assay for detection of Wuchereria bancrofti DNA in blood and mosquitoes. *Am J Trop Med Hyg* 2006;74(5):826-32.
